# *SRSF2* mutations drive daunorubicin resistance in acute myeloid leukemia via *THBS1* stabilization

**DOI:** 10.1186/s13046-026-03649-y

**Published:** 2026-01-30

**Authors:** Wu Ye, Xia Wu, Yuqian Tang, Ying Zhang, Yiwen Du, Kun Yang, Yankun Yang, Yuping Gong

**Affiliations:** 1 Department of Hematology, West China Hospital, Sichuan University, No.37 GuoXue Xiang, Chengdu, 610041 Sichuan Province China; 2 National-Local Joint Engineering Research Center of Biodiagnostics & Biotherapy, The Second Affiliated Hospital of Xi’an Jiaotong University, Xi’an, Shaanxi Province 710004 China

**Keywords:** Acute myeloid leukemia, *SRSF2* mutations, Chemotherapy resistance, Cellular metabolism, Synergistic effect

## Abstract

**Background:**

Acute myeloid leukemia (AML) is an aggressive hematologic malignancy characterized by the uncontrolled growth of immature myeloid cells, often with a poor prognosis due to therapy resistance. This study investigated the prognostic significance of *SRSF2* mutations in AML and their impact on chemotherapeutic drug sensitivity.

**Methods:**

The prognostic value of *SRSF2* mutations was analyzed in AML patients. *SRSF2*-mutant cell models were generated via lentiviral transduction for drug sensitivity testing. Xenograft mice were used to assess daunorubicin (DNR) efficacy. Mechanistic studies included transcriptomics, splicing analysis, mRNA stability, polysome profiling, RNA immunoprecipitation, and metabolic assays to identify targetable resistance pathways.

**Results:**

Clinical analysis revealed that *SRSF2* mutations decreased the survival of AML patients. *In vitro* experiments demonstrated that *SRSF2* mutation reduced the sensitivity of AML cells to drugs such as DNR and homoharringtonine but did not affect the response to venetoclax. In mouse models, DNR treatment was effective against wild-type AML but showed significantly reduced efficacy in suppressing tumors and improving survival in *SRSF2*-mutant AML. Mechanistically, *SRSF2* mutation impaired the interaction between the *SRSF2* protein and *THBS1* mRNA, prolonging the *THBS1* mRNA half-life and enhancing its translation efficiency, leading to THBS1 protein accumulation. Additionally, the mutation altered the splicing pattern of *ETV7* and upregulated its expression, potentially mediating DNR resistance. Metabolic analysis revealed that mutant cells presented increased spare respiratory capacity, supporting energy demands under stress. Inhibition of the PDGFB pathway (CP-673451) synergistically enhanced the cytotoxic effect of DNR on mutant cells.

**Conclusions:**

*SRSF2* mutations promoted DNR resistance through multiple mechanisms, and targeted combination therapy with PDGFB pathway inhibitors may represent a novel strategy to improve therapeutic outcomes in patients with mutations.

**Supplementary Information:**

The online version contains supplementary material available at 10.1186/s13046-026-03649-y.

## Introduction

Acute myeloid leukemia (AML) is an aggressive form of cancer that originates in the bone marrow, where abnormal white blood cells called myeloblasts proliferate uncontrollably [1]. These malignant cells disrupt the production of healthy blood cells, leading to symptoms such as fatigue, frequent infections, easy bruising, and anemia [2]. The diagnosis of AML typically involves a comprehensive assessment, including clinical presentation, cell morphology, immunophenotyping, genetic analysis, and molecular biology characteristics [3]. With the rapid development of molecular biology and genomics, the World Health Organization has continuously updated the diagnostic criteria for AML, with an increasing focus on genetic and molecular driving factors [4, 5].The International Consensus Classification (ICC) of Myeloid Neoplasms and Acute Leukemias (ICC-2022) recognizes TP53-mutated myeloid neoplasms as a new category, including AML with TP53 mutation [6]. Additionally, the presence of recurrent genetic abnormalities, such as *PML*::*RARA*, *CBFβ*::*MYH11*, *MLLT3*::*KMT2A*, *MECOM* rearrangements, and mutations in *NPM1* and bZIP *CEBPA*, along with ≥ 10% blasts in the bone marrow or peripheral blood, meets the diagnostic criteria for AML [7]. With a deeper understanding of the pathogenesis, molecular mechanisms, and genetic basis of AML, treatment strategies have advanced significantly, and the development of targeted therapies has provided patients with more treatment options and improved outcomes [8].

The splicing of pre-mRNAs plays a pivotal role in modulating gene expression. Mutations in RNA splicing factors, such as *SRSF2*, *SF3B1*, and *U2AF1*, are commonly found in patients with MDS and AML [9]. SRSF2 contains an RNA recognition motif (RRM) and an arginine/serine (RS)-rich domain [10]. The RRM exhibits sequence-specific binding to RNA, whereas the RS-rich domain predominantly facilitates protein-protein interactions and plays a critical role in orchestrating spliceosome assembly and disassembly [11]. During normal physiological processes, SRSF2 recognizes and binds to cis-regulatory elements on pre-mRNAs, facilitating the recruitment of additional splicing factors to ensure accurate regulation of alternative splicing [12]. *SRSF2* regulates gene expression patterns through alternative splicing, maintaining homeostasis in hematopoiesis, liver metabolism, and the development of skeletal muscle and bone marrow [13–16].Among the mutational sites of *SRSF2*, the frequency of mutations in the codon at position 95 is the highest [17], with P95H, P95R, and P95L being the most common mutations. Multiple studies have demonstrated that *SRSF2* mutations alter the binding and splicing characteristics of *SRSF2* with target RNA, leading to genome-wide splicing changes and gene expression dysregulation [18]. This may result in the production of loss-of-function isoforms of certain tumor suppressor genes or gain-of-function isoforms of oncogenes, which interfere with the normal regulation of multiple signaling pathways in cells, thereby contributing to the onset and progression of the disease [19].

*SRSF2* mutations confer an adverse prognosis in AML patients and are significantly associated with older age and elevated blast counts [20]. Notably, they facilitate rapid progression from low-risk MDS to AML [21], substantially accelerating leukemic transformation [22]. Multivariate analysis revealed *SRSF2* mutation as an independent poor prognostic factor in elderly AML patients who did not receive intensive therapy [23]. Compared with those in de novo AML, *SRSF2* mutations exhibit > 95% specificity in secondary AML (s-AML), indicating that this mutation is a “secondary-type” mutation. These mutations are correlated with adverse clinical outcomes, including lower complete remission rates, higher reinduction frequency, and reduced event-free survival. Persistent *SRSF2* mutations are associated with increased cumulative relapse rates [24], and patients with residual mutations during remission are typically older and exhibit shorter relapse-free and overall survival (OS) [25]. Approximately 45% of s-AML patients with *SRSF2* mutations also harbor *ASXL1* mutations [26]. Compared with patients harboring a single mutation, *ASXL1*/*SRSF2* comutated patients are older [27], show delayed platelet recovery postinduction chemotherapy [28], and exhibit dismal OS without long-term survival, suggesting synergistic adverse effects [29]. Comutations of *SRSF2* and *RUNX1* also predict poor outcomes in patients with AML [30]. Despite advances in understanding the pathogenic role, clinical implications, and prognostic impact of *SRSF2* mutations in AML, critical questions remain unresolved, particularly their contribution to therapy resistance. Further investigations are warranted to develop targeted therapies and improve survival in this high-risk population.

## Methods

### Clinical data analysis

We collected clinical data (including baseline characteristics and genetic mutations) from patients with myeloid malignancies who underwent genetic testing at West China Hospital, Sichuan University between June 2018 and September 2023. AML patients were matched at a 2:1 ratio using propensity score matching (PSM) to minimize confounding bias. Survival analysis was performed via the Kaplan-Meier method, and intergroup survival differences were assessed via weighted Cox proportional hazards regression. PSM balances covariates by matching patients with similar propensity scores, reducing selection bias and approximating randomized trial conditions for more reliable effect estimation [31].

### Cell culture

The cells were maintained in medium supplemented with 10% fetal bovine serum (FBS). Specifically, 293T cells were cultured in DMEM, HEL and Kasumi-1 cells in RPMI 1640, and BaF3 cells were cultured in RPMI 1640 containing 1 ng/mL IL-3 (final concentration). All cell lines were incubated at 37 °C with 5% CO₂.

### Generation of stable SRSF2-mutant (SRSF2^mut^) cell lines

HEL, Kasumi-1, and BaF3 cells were revived and cultured to optimal growth conditions. On the day of transduction, 2 × 10⁵ cells per well were seeded in 6-well plates (3 wells each for HEL and BaF3, 2 wells for Kasumi-1) in 1 mL fresh medium and incubated for 2 h. Subsequently, 1 mL of thawed lentivirus was added to each well: HEL and BaF3 cells were transduced with Lvx-ev-vector, Lvx-ev-SRSF2^mut^, or Lvx-ev-SRSF2^mut/flag^, whereas Kasumi-1 cells received Tet-on-vector or Tet-on-SRSF2^mut^. Polybrene (1.6 µL, 8 mg/mL) was added to each well to increase the transduction efficiency. After 48 h, the medium was replaced with 2 mL of fresh medium containing puromycin (final concentration of 1 µg/mL) for selection.

Two weeks later, transduced HEL and BaF3 cells were collected to verify SRSF2 and flag expression. For Kasumi-1 cells, 2 × 10⁵ selected cells per well were plated in 6-well plates and treated with doxycycline hyclate (Dox) for 72 h to induce SRSF2^mut^ expression before analysis.

This process successfully generated the following stable cell lines: HEL control (HEL-CTR) cells, SRSF2^mut^-expressing HEL (HEL-SRSF2^mut^) cells, SRSF2^mut/flag^- expressing HEL (HEL-SRSF2^mut/flag^) cells, BaF3 control (BaF3-CTR) cells, SRSF2^mut^-expressing BaF3 (BaF3-SRSF2^mut^) cells, SRSF2^mut/flag^-expressing BaF3 (BaF3-SRSF2^mut/flag^) cells, Kasumi-1 control (Kasumi-1-CTR) cells, and Dox-induced SRSF2^mut^-expressing Kasumi-1 (Kasumi-1-SRSF2^mut^) cells.

### Generation of HEL cell lines stably expressing luciferase

HEL-CTR and HEL-SRSF2^mut^ cells (2 × 10⁵ cells per well) were seeded in 6-well plates containing 2 mL of RPMI 1640 medium, followed by the addition of 6 µL of luciferase-expressing lentivirus and 1.6 µL of polybrene (8 mg/mL). After 48 h of incubation, the medium was replaced with fresh medium containing blasticidin (BSD) at a final concentration of 2 µg/mL for selection, and the medium was subsequently changed such that the BSD concentration was maintained while puromycin selection was continued throughout the culture period.

Two weeks after selection, the cells were harvested and resuspended in 1x phosphate-buffered saline (1x PBS) for luminescence assays. The cell suspensions (5 × 10⁵ cells/100 µL) were transferred to opaque 96-well plates, with parallel samples treated with 2 µL of potassium luciferin (15 mg/mL) or 1x PBS as a negative control. Following a 30-minute incubation in the dark, bioluminescence was quantified via a multimode microplate reader to confirm successful luciferase reporter integration in both the HEL-CTR and HEL-SRSF2^mut^ cell lines.

### Cell growth curve

The cells were seeded in 12-well plates at 5 × 10⁴ cells per well in 1 mL of complete RPMI 1640 medium without puromycin (*n* = 3 replicates/group). The cells were cultured at 37 °C with 5% CO₂ (designated day 0). Viable cell counts were performed daily from days 1 to 4, and growth curves were plotted on the basis of the quantified data.

### Cytotoxicity assay

The cells were seeded in 96-well plates at 1 × 10⁴ per well (HEL/BaF3) or 1.5 × 10⁴ per well (Kasumi-1) in 90 µL complete medium, with 100 µL medium-only wells used as blanks. Drug treatments (10 µL/well) included daunorubicin hydrochloride (DNR), cytarabine (Ara-C), homoharringtonine (HHT), azacitidine (AZA), decitabine (Dec), and venetoclax (Ven) at graded concentrations, with control wells receiving medium only (*n* = 3 replicates, total volume 100 µL/well). After 48 h of incubation, 20 µL of MTT (5 mg/mL) was added to each well. After 4–6 h of formazan formation, 100 µL solubilization buffer was added, and the plates were incubated overnight. The absorbance (570 nm) was measured to calculate cell viability.

### Apoptosis assay

HEL cells were seeded in 6-well plates at 3 × 10⁵ cells per well in 2 mL medium containing either vehicle or drugs at the following final concentrations: 4 µM DNR, 500 nM HHT, 400 nM AZA, 500 nM Ara-C, 2 µM Dec, or 15 µM Ven, with triplicate wells per group. Dox-induced Kasumi-1 cells were plated at 3 × 10⁵ cells per well and treated with 800 nM DNR or 10 nM HHT (final concentrations). BaF3 cells were seeded at 2 × 10⁵ cells per well and exposed to 1 µM DNR or 25 nM HHT (final concentrations). After 48 h of incubation, the cells were harvested and washed twice with ice-cold 1x PBS. The washed cells were collected by centrifugation, and 100 µL of 1x binding buffer was added to each tube under light-protected conditions. Subsequently, 1 µL of Annexin V-647 and 0.5 µL of 7-AAD were added to each tube (for the DNR-treated Kasumi-1 and BaF3 cells, only 1 µL of Annexin V-647 was added to each tube). The mixture was gently pipetted to homogenize and incubated for 15 min in the dark. Then, 200 µL of 1x binding buffer was added to each tube, followed by gentle flicking to mix the contents. Apoptosis was analyzed by flow cytometry (FCM).

### Cell cycle assay

HEL cells were seeded at 3 × 10⁵ cells per well in 6-well plates. The cells were treated with drugs or solvent diluted in complete RPMI 1640 medium, with a final volume of 2 mL per well. The final drug concentrations used were 400 nM HHT, 300 nM Ara-C, 10 µM Ven, 200 nM AZA, and 1.5 µM Dec. The untreated control group and AZA-treated group were harvested after 24 h, whereas the HHT-, Ara-C-, Ven-, and Dec-treated groups were harvested after 48 h. Kasumi-1 cells were seeded at 3 × 10⁵ cells per well in 6-well plates. The cells were treated with HHT or solvent diluted in complete RPMI 1640 medium, with a final volume of 2 mL per well and a final HHT concentration of 10 nM. The cells were harvested after 24 h of incubation. BaF3 cells were seeded at 2 × 10⁵ cells per well in 6-well plates. The cells were treated with HHT or solvent diluted in complete RPMI 1640 medium, with a final volume of 2 mL per well and a final HHT concentration of 25 nM. The cells were harvested after 24 h of incubation.

Following overnight fixation in 70% ethanol at 4 °C, the cells were stained with propidium iodide (PI) working solution and incubated at 37 °C for 30 min in the dark. The cell suspensions were filtered through 40 μm nylon mesh prior to analysis. The cell cycle distribution was determined by FCM.

### AML xenograft mouse model

Female NOD/SCID gamma mice (6–8 weeks old) were intravenously injected with 1 × 10^6^ luciferase-expressing HEL-CTR or HEL-SRSF2^mut^ cells via the tail vein. Tumor engraftment was confirmed on day 8 postinjection via in vivo imaging system (IVIS). The mice were stratified into four groups (*n* = 6/group) with matched baseline tumor burdens on the basis of IVIS: CTR, CTR + DNR, SRSF2^mut^ and SRSF2^mut^ + DNR. Therapeutic intervention was initiated on day 9 postinjection. The treatment groups received 4 mg/kg DNR via tail vein injection every 48 h for three consecutive doses, whereas the control groups were administered equivalent volumes of normal saline following the same schedule. Mouse body weights and survival rates were recorded throughout the study period. The tumor burden was dynamically monitored via IVIS. Bioluminescence signals were quantified via IVIS software. Survival curves were generated via Kaplan-Meier analysis, with between-group differences assessed via the log-rank test.

### RNA sequencing

DNR was first diluted to 4 µM in complete medium and vortexed thoroughly. The control groups received equivalent volumes of 1× PBS. HEL cells were plated at a density of 1 × 10⁶ cells per 6-cm dish in 2 mL of treatment medium, with the volume subsequently increased to 4 mL using fresh complete medium to yield a final DNR concentration of 2 µM. The experimental design included four conditions (CTR, SRSF2^mut^, CTR + DNR, SRSF2^mut^ + DNR), with three biological replicates per condition. Following 16 h of incubation, the cells were pelleted via centrifugation, and the medium was removed. The pellets were washed twice with 1× PBS. A total of 400 µL of TRIzol reagent was added to each pellet via vigorous pipetting, followed by immediate flash freezing in liquid nitrogen. Frozen samples were maintained at -80 °C until RNA sequencing (Illumina platform) was performed by Novogene. Enrichment analysis of the differentially expressed genes (DEGs) was performed via WikiPathways, Kyoto Encyclopedia of Genes and Genomes (KEGG), Reactome, Gene Ontology (GO), and the Molecular Signatures Database (MSigDB). Alternative splicing analysis was conducted via rMATS software.

### Investigation of cell signaling pathways (at the mRNA level)

HEL cells were seeded in 6-well plates at a density of 3 × 10^5^ cells per well. Complete medium containing either the drug solvent or DNR was added to each well, with a final volume of 2 mL per well. The final concentration of DNR was 4 µM. Following 16 h of incubation, the cells were harvested. The cell pellets were collected by centrifugation after two washes with ice-cold 1× PBS. RNA extraction, reverse transcription, and quantitative PCR (qPCR) were performed according to the manufacturers’ protocols. The sequences of primers used in this study are provided in Supplemental Table 1.

### Investigation of cell signaling pathways (at the protein level)

For HEL cells, cells were seeded at 1 × 10⁶ cells per 6-cm dish in 2 mL of complete medium containing either solvent or DNR. The total volume was adjusted to 4 mL with fresh complete medium to achieve a final DNR concentration of 2 µM. For Kasumi-1 or BaF3 cells, cells were seeded at 1 × 10⁶ cells per 6-cm dish in 2 mL of complete medium containing either solvent or DNR. The total volume was adjusted to 4 mL with fresh complete medium to achieve a final DNR concentration of 250 nM. After 16 h of incubation, the cells were collected via centrifugation, washed twice with ice-cold 1× PBS, and pelleted.

An additional set of HEL cells was plated and treated with DNR at the same concentration for 16 h. After collection by centrifugation, the supernatant was discarded, and the cells were resuspended in fresh complete medium. The entire cell suspension was then replated into culture dishes. Monensin (prediluted in medium) was added to achieve a final volume of 4 mL per dish with a final concentration of 2 µM. Following a 3-hour incubation, the cells were collected via centrifugation, washed twice with ice-cold 1× PBS, and pelleted.

Protein expression levels were analyzed via Western blotting (WB) for the following targets: THBS1, IL1B, PDGFB, JUP, AQP1, GJB2, BIRC3, RelB, ETV7, MCL1, BCL-XL, BCL2, BAX, PUMA, MYC, β-actin, Mcl1, Bcl2, Bax, cyclin D1, and eIF4E.

### RNA stability assay

Under light-protected conditions, actinomycin-D was diluted to 10 µg/mL in complete medium. HEL cells were seeded at 1 × 10⁶ cells per 6-cm dish, with five replicates prepared for each group. Each dish received 2 mL of the prepared actinomycin-D-containing medium, followed by adjustment to a final volume of 4 mL with fresh complete medium, yielding a final actinomycin-D concentration of 5 µg/mL. After gentle mixing, the dishes were incubated under standard culture conditions. The cells were harvested at 0, 1, 3, 6, and 9 h posttreatment. The cells were washed with prechilled 1× PBS, and the pelleted cells were collected. Then, 400 µL of cRL1 from the Forgene RNA extraction kit was added, and the cells were fully lysed by mixing. The lysates were stored at − 80 °C for subsequent RNA extraction from cells collected at different time points. Reverse transcription and qPCR were subsequently performed. Using the 0-hour time point as the control and *GAPDH* as the internal reference, the normalized mRNA expression levels of each gene in HEL cells at different time points were calculated. The mRNA half-lives of the genes were analyzed, and the data were plotted via GraphPad Prism.

### RNA Immunoprecipitation (RIP)

For both HEL-CTR and HEL-SRSF2^mut^, 2 × 10⁷ cells were collected and washed twice with 2 mL of ice-cold 1× PBS. After centrifugation, the cell pellets were resuspended in 400 µL of complete RIP lysis buffer and pipetted 10 times to ensure complete lysis. The RIP assay was performed according to the manufacturer’s protocol (Sangon Biotech RIP Kit) including sample preparation, magnetic bead preparation, antibody incubation, and immunoprecipitation steps. RNA obtained from RIP was extracted via the TRIzol method, followed by reverse transcription into cDNA. The Ct values of the *THBS1* gene in each group were determined via qPCR.

The fold enrichment was calculated relative to that of IgG via the following formula:

Fold enrichment = 2^(-∆∆Ct), where ∆∆Ct = (Ct_IP_-Ct_Input_)-(Ct_IgG_-Ct_Input_).

### Polysome profiling

#### Sucrose gradient preparation

Five milliliters of 10× sucrose gradient buffer was prepared as follows: 200 mM HEPES (pH 7.6), 1× protease inhibitor cocktail (EDTA-free), 1 M KCl, 100 µg/mL cycloheximide (CHX), 50 mM MgCl₂, and 100 U/mL RNase inhibitor [32]. Five milliliters of 10× sucrose gradient buffer was diluted to 50 mL of 1× buffer in nuclease-free water. A 60% (w/v) sucrose solution (50 mL) was prepared with nuclease-free water and then diluted to 5% and 50% sucrose solutions (40 mL each) via 1× sucrose gradient buffer. All the solutions were filtered through a 0.22 μm membrane to prevent tubing clogging during fractionation. The half-full point of each ultracentrifuge tube (2 tubes total) was marked via the gradient marker indicator block. Using a layering device, 5% sucrose solution was added until 2 mm above the half-full mark, followed by underlaying with 50% sucrose solution until the liquid interface reached the half-full point. The tubes were sealed (using long caps for isopycnic centrifugation), placed symmetrically in the gradient preparation rotor, and centered on the gradient maker’s magnetic disk for stabilization. The gradient maker was run to generate a linear 5%-50% gradient.

#### Polysome isolation and sedimentation

HEL-CTR and HEL-SRSF2^mut^ cells were prepared under optimal growth conditions, with the culture medium replaced one day prior to experimentation. Each cell culture dish was supplemented with 10 mL of fresh complete medium. On the day of use, 100 µL of 10 mg/mL CHX was added per dish (final 100 µg/mL), followed by 5 min of incubation. The cells were harvested (200 × g, 4 °C, 5 min) and washed 3 times with 5 mL of ice-cold 1× PBS containing 100 µg/mL CHX. After centrifugation, the cell pellets were resuspended in 425 µL of hypotonic buffer (5 mM Tris-HCl, 1.5 mM KCl, 2.5 mM MgCl₂, and 1× protease inhibitor cocktail), supplemented with 5 µL of 10 mg/mL CHX, 100 U of RNase inhibitor, and 1 µL of 1 M dithiothreitol (DTT), vortexed for 5 s. Next, 25 µL of 10% sodium deoxycholate (final 0.5%) and 25 µL of 10% Triton X-100 (final 0.5%) were added, followed by vortexing for 5 s [32]. The lysates were centrifuged (16,000 rpm, 4 °C, 7 min), and ~ 500 µL of the supernatant was transferred to prechilled 1.5 mL tubes. A 50 µL aliquot of lysate was collected as input for cytoplasmic steady-state mRNA determination. To the input tube, 700 µL of nuclease-free water was added, followed by the addition of 750 µL of TRIzol reagent. The mixture was immediately flash-frozen in liquid nitrogen and stored at -80 °C for subsequent use. The ultracentrifuge tubes were carefully placed in rotor buckets, and a 200 µL sucrose gradient was removed from the top. The lysates were loaded onto the gradient surface. The tubes were weighed and balanced before ultracentrifugation. Centrifugation was performed using an SW40Ti rotor (36,000 rpm, 4 °C, 2 h).

#### Polysome fractionation and RNA extraction

Postcentrifugation gradients were carefully retrieved and placed in adapters. The ultracentrifuge tubes were placed on a UV monitor (254 nm), and 17 nuclease-free 2 mL tubes were arranged in a fraction collector. The pump was set to 1.5 mL/min, with 30 s collection intervals (750 µL/fraction). Fractions were collected on the basis of UV absorbance profiles, and peaks were recorded. Each fraction was mixed with 750 µL of TRIzol and flash-frozen in liquid nitrogen. RNA was extracted from all fractions and input samples via the TRIzol method, reverse-transcribed into cDNA, and analyzed via qPCR. The mRNA levels of genes in each fraction were normalized to the input control using *GAPDH* as the internal reference.

### Alternative splicing analysis of ETV7 

HEL cells were seeded at 3 × 10⁵ cells per well in 6-well plates. Drug solvent or DNR diluted in medium was added to each well, with the final volume adjusted to 2 mL and the final DNR concentration set at 4 µM. After 16 h of incubation, the cells were collected via centrifugation, washed twice with ice-cold 1× PBS, and pelleted. Cellular RNA was extracted via the TRIzol method, reverse-transcribed into cDNA, and analyzed via qPCR. The ETV7 primers (Supplemental Table 2) were designed to detect all known *ETV7* splice variants. The forward primer of S-ETV7 specifically targets exon 2 of *ETV7*; therefore, amplification of this variant would not occur if exon 2 were skipped. The mRNA levels of ETV7 and S-ETV7 were normalized to those of HEL-CTR and *GAPDH* (internal reference).

### Histopathological analysis and detection of CD45 + cells in mouse organs

#### Histopathological analysis of mouse organs

Luciferase-labeled HEL-CTR and HEL-SRSF2^mut^ cells (1 × 10⁶ cells per mouse) were transplanted into mice via tail vein injection to establish a tumor model. On day 8 posttransplantation, the tumor burden was assessed via IVIS, and the mice were divided into 4 groups (3 mice per group). Therapeutic intervention was initiated on day 9 posttransplantation. The treatment groups received 5 mg/kg DNR via tail vein injection every 48 h for three consecutive doses, whereas the control groups were administered equivalent volumes of normal saline following the same schedule. All the mice were euthanized on day 26 posttransplantation. The lower limb bones, livers, spleens, and lungs were harvested from the mice. After being weighed, portions of the liver and spleen, along with one lower limb bone and lung tissue, were fixed in 4% paraformaldehyde for a minimum of 24 h. Fixed lower limb bones were decalcified in decalcification solution until optimal tissue softness and elasticity were achieved. These visceral tissues were subsequently processed for hematoxylin and eosin (H&E) staining and immunohistochemical (IHC) staining analysis.

#### Detection of CD45 + cells in mouse organs

Small portions of the liver, spleen, and one lower limb bone were separately homogenized in ice-cold 1× PBS, filtered, and centrifuged. The supernatants were discarded, and the pellets were retained. Each pellet was resuspended in 1 mL RBC lysis buffer, incubated at room temperature for 5 min, washed with 2 mL 1×PBS and centrifuged to retain the pellets. The pellets were resuspended in 100 µL of 1× PBS, stained with 1 µL of CD45-APC-Cy7 for 30 min, washed with 1 mL of 1× PBS, and finally resuspended in 300 µL of 1× PBS. CD45 + cell populations were quantified via FCM.

### Mitochondrial stress test

The assay was performed via the Seahorse XFe24 system. The sensor cartridge was hydrated with XF calibrant solution (1 mL/well) and incubated overnight at 37 °C without CO₂. Stock solutions of FCCP (1 mM), oligomycin (15 mM), and Rot/AA (5 mM) were prepared in ethanol and stored at -80 °C. Seahorse assay medium was prepared by supplementing RPMI 1640 with 2 mM glutamine, 1 mM pyruvate, and 10 mM glucose.

HEL cells were plated at a density of 1 × 10⁵ cells per well in poly-L-lysine-coated XFe24 plates (3 wells per group). Following centrifugation (200 × g, 1 min, no brake) and initial incubation (37 °C, 30 min), 400 µL of preequilibrated assay medium was carefully added to each well. The plates were incubated for an additional 25 min at 37 °C prior to assay initiation. Drug working solutions (10×) were prepared as specified in Supplemental Tables 3 and loaded into the corresponding ports of the prehydrated sensor cartridge.

The Seahorse XFe24 system was initiated, and the “Cell Mito Stress Test” program was selected. After the experimental groups and background correction wells were configured, the hydrated sensor cartridge and calibration plate were properly positioned on the tray. Following system calibration, the calibration plate was replaced with a cell culture plate as prompted. The automated analysis and data acquisition phase was initiated, with a total run time of approximately 100 min.

### Glycolytic rate assay

The glycolytic rate assay followed the same preparatory steps as the mitochondrial stress test prior to reagent preparation. 2-Deoxy-D-glucose (2-DG) was diluted to 500 mM, and Rot/AA was diluted to 5 mM in assay buffer for drug preparation. A 10x working solution was prepared according to Supplemental Table 4, followed by sequential addition of both compounds to the wells. Instrumental analysis was performed via the “Glycolytic Rate Assay” program.

### Synergistic cytotoxic effects of DNR and CP-673451

For HEL cells, drug solutions were prepared by serial 1:1 dilution in complete medium. DNR was initially prepared at 16 µM (1 mL total volume), then 500 µL of DNR solution was mixed with 500 µL of fresh medium to generate 8 µM, and this step was repeated to obtain 4 µM and 2 µM solutions. Similarly, CP-673451 was initially prepared at 20 µM and serially diluted to concentrations of 10, 5, 2.5, and 1.25 µM. In the 96-well plate setup, 25 µL of each DNR dilution (16, 8, 4, and 2 µM) was added to the vertical columns, while 25 µL of each CP-673451 dilution (20, 10, 5, 2.5, and 1.25 µM) was added to the horizontal rows. The control wells received 25 µL of complete medium alone. After drug addition, 50 µL of cell suspension (1 × 10^4^ cells) was added to each well, resulting in final DNR concentrations of 0, 0.5, 1, 2, and 4 µM and final CP-673451 concentrations of 0, 0.3125, 0.625, 1.25, 2.5, and 5 µM in a total volume of 100 µL per well.

For Kasumi-1-SRSF2^mut^ cells, DNR was initially prepared at 2000 nM and serially diluted to concentrations of 1000 and 500 nM, while CP-673451 was initially prepared at 40 nM and diluted to concentrations of 20 and 10 nM. In the 96-well plate setup, 25 µL of each DNR dilution was added to horizontal rows, and 25 µL of each CP-673451 dilution was added to vertical columns. Following drug addition, 50 µL of cell suspension (2 × 10^4^ cells) was added per well, yielding final DNR concentrations of 0, 125, 250, and 500 nM and final CP-673451 concentrations of 0, 2.5, 5, and 10 nM in a 100 µL total volume.

All the plates included blank wells containing 100 µL of complete medium without cells. After 48 h of incubation, cell viability was measured using the MTT assay.

Finally, the Synergy Finder platform was used to evaluate the synergistic effects of the two drugs, employing both the zero interaction potency (ZIP) and highest single agent (HSA) models. The ZIP model compares the observed combination effect with the expected effect assuming no interaction between the drugs, effectively eliminating interference from dose-response curves. The HSA model compares the observed combination effect with the best effect of the two single agents at corresponding concentrations, providing a conservative estimate of synergy. Higher synergy scores calculated by both models indicate stronger synergistic effects, with a score greater than 10 generally considered to represent a significant synergistic effect.

### Synergistic apoptotic effects of DNR and CP-673451

HEL cells were seeded at 3 × 10⁵ cells per well in 6-well plates and treated with either vehicle control or drugs diluted in culture medium. The single-agent groups were exposed to final concentrations of 4 µM DNR or 3 µM CP-673451, whereas the combination group received both 4 µM DNR and 3 µM CP-673451 simultaneously. Similarly, Kasumi-1 cells were seeded at 3 × 10⁵ cells per well in 6-well plates and treated with either vehicle control or drugs. The single-agent groups were exposed to final concentrations of 500 nM DNR or 10 nM CP-673451, whereas the combination group was treated with both 500 nM DNR and 10 nM CP-673451 concurrently. Apoptosis was analyzed by FCM after 48 h of incubation.

### Soft agar colony formation assay

We performed a soft agar colony formation assay to evaluate the synergistic inhibitory effects of DNR and CP-673451. First, a 2% agarose solution was prepared by dissolving 1 g of agarose in 50 mL of ddH2O, autoclaved, and stored at room temperature (RT). Prior to use, the solution was microwaved intermittently until transparent and cooled to ~ 50 °C. Simultaneously, 50 mL of 15% FBS-supplemented culture medium was prepared, aliquoted into 3 mL and 5 mL portions in 15 mL tubes, and prewarmed at 42 °C. For the base layer, 3 mL of medium was mixed with 1 mL of 2% agarose (0.5% final) and plated at 1 mL/well in 6-well plates (*n* = 3 per group). After solidification at RT, the top layer was prepared by mixing 5 mL of medium with 1 mL of 2% agarose (0.33% final) containing either HEL or Kasumi-1 cells (Dox-induced for Kasumi-1), yielding 3,000 HEL or 4,000 Kasumi-1 cells per well. Following solidification, 1 mL of culture medium containing 1 µg/mL puromycin (plus 100 ng/mL Dox for Kasumi-1) was added per well (day 0).

On day 5, the top layer of culture medium was carefully aspirated. Drugs were prepared in culture medium to achieve the following final concentrations: for HEL cells, single-agent wells contained either 2 µM DNR or 1 µM CP-673451, while combination wells received both 2 µM DNR and 1 µM CP-673451, with control wells treated with vehicle only; for Kasumi-1 cells, single-agent wells contained either 200 nM DNR or 5 nM CP-673451, while combination wells received both 200 nM DNR and 5 nM CP-673451, with control wells similarly treated with vehicle. Subsequently, 1 mL of the drug-medium mixture was added to each designated well, and the cells were cultured until day 12 with medium replacement every 3–4 days. On day 12, the top layer of medium was replaced with fresh drug-free medium in all wells. By day 14, the cell clones were stained with 300 µL crystal violet after medium removal. After being stained and incubated for 3 min at RT, the samples were washed multiple times with 1× PBS until clear morphological visualization of the clones was achieved. Colony formation was documented photographically and analyzed using ImageJ-based quantification.

### Statistical analysis

Statistical analysis was conducted via GraphPad Prism 8.0, SPSS Statistics 25, and R (v4.3.1). Continuous variables were analyzed as follows: unpaired Student’s t test for normally distributed data between two groups; one-way ANOVA for multiple group comparisons; and the Mann-Whitney U test for nonnormally distributed data. Categorical variables were assessed via the χ² test or Fisher’s exact test. Survival analysis was performed via the Kaplan-Meier method, with intergroup differences assessed via the log-rank test. In propensity score-matched cohorts (2:1 ratio), survival outcomes were evaluated through Kaplan-Meier analysis coupled with a weighted Cox proportional hazards regression model. The threshold for statistical significance was set at *P* < 0.05, with asterisks denoting specific significance levels: *P < 0.05, **P < 0.01, ***P < 0.001, and ****P < 0.0001.

## Results

### Prognostic significance of *SRSF2* mutations in AML

We conducted a retrospective analysis of patients who received genetic testing at West China Hospital between June 2018 and September 2023. Among this cohort, *SRSF2* mutations were identified in 99 patients. These mutations spanned nine unique sites, including c.1G > A, c.130T > C (p.Tyr44His), c.161 C > T (p.Ser54Phe), c.281_283dup (p.Arg94dup), c.284_304del (p.Pro95_Ser101del), c.284_307del (p.Pro95_Arg102del), c.284 C > A (p.Pro95His), c.284 C > G (p.Pro95Arg), and c.284 C > T (p.Pro95Leu), and were predominantly located in the interdomain region between the RRM and RS domains. The analysis focused on 36 treatment-naïve non-M3 AML patients from this SRSF2^mut^ cohort, with the following French-American-British subtypes: M2 (*n* = 23), M4 (*n* = 8), M5 (*n* = 3), M1 (*n* = 1), and M0 (*n* = 1). Given the advanced age and numerous cooccurring mutations in these patients, known prognostic factors in AML, we performed PSM (2:1) against wild-type *SRSF2* controls (*n* = 68) from our non-M3 AML database. The matching parameters included sex, age, and critical molecular markers (*NPM1* mutation, *CEBPA* biallelic mutation, *TP53* mutation, and *FLT3*-ITD) (Supplemental Table 5). The matched cohorts demonstrated comparable baseline hematologic parameters, including white blood cell counts, hemoglobin levels, platelet counts, and blast percentages in both peripheral blood and bone marrow (Supplemental Fig. 1A). Survival analysis initially revealed a borderline nonsignificant trend toward shorter survival in SRSF2^mut^ patients than in their wild-type counterparts (HR 1.40, 95% CI 0.84–2.33; *P* = 0.195) (Supplemental Fig. 1B). Notably, this association became statistically significant when the analysis was restricted to nonhematopoietic stem cell transplantation (non-HSCT) patients, with mutant patients showing markedly inferior survival outcomes (HR 1.70, 95% CI 1.01–2.86; *P* = 0.046) (Supplemental Fig. 1C).

### *SRSF2* mutation conferred altered molecular phenotypes in AML cells

**Fig. 1:**
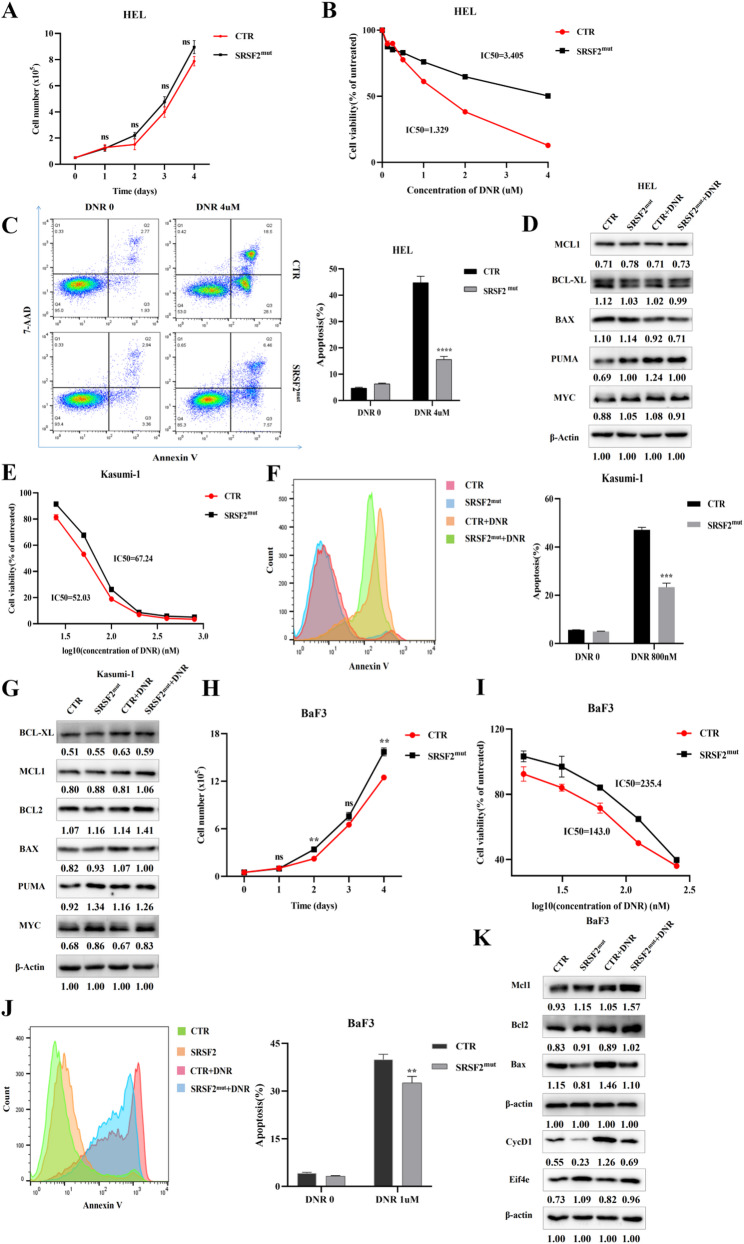
Impact of *SRSF2* mutation on cellular proliferation and the DNR response. (**A**) Growth curve of HEL cells. (**B**) Viability assessment of DNR-treated HEL cells. (**C**) Apoptosis quantification in DNR-exposed HEL cells. (**D**) WB analysis of cell cycle-related and apoptosis-related proteins in HEL cells. (**E**) Viability assessment of DNR-treated Kasumi-1 cells. (**F**) Apoptosis quantification in DNR-exposed Kasumi-1 cells. (**G**) WB analysis of cell cycle and apoptosis regulatory proteins in Kasumi-1 cells. (**H**) Growth curve of BaF3 cells. (**I**) Viability assessment of DNR-treated BaF3 cells. (**J**) Apoptosis quantification in DNR-exposed BaF3 cells. (**K**) WB analysis of cell proliferation- and apoptosis-associated proteins in BaF3 cells. Abbreviations: DNR, daunorubicin hydrochloride; WB, western blotting

To elucidate the mechanisms of *SRSF2* mutations in AML, we engineered HEL cells to stably express SRSF2^P95H^, the most prevalent variant in AML. Successful expression was confirmed at both the transcriptional and translational levels via mutation-specific qPCR and anti-Flag immunoblotting following puromycin selection (Supplemental Fig. 2). Comparative analysis revealed distinct drug response patterns between SRSF2 mutant and wild-type HEL cells. Proliferation assays revealed similar growth curves between the CTR and SRSF2^mut^ cells (Fig. 1A). MTT assays demonstrated that DNR sensitivity was lower in SRSF2^mut^ cells (IC50 = 3.405 µM) than in CTR cells (IC50 = 1.329 µM) (Fig. 1B). Following 48 h of DNR treatment, the percentage of apoptotic SRSF2^mut^ cells (15.6%) was significantly lower than that of CTR cells (44.8%) (Fig. 1C). WB analysis showed that PUMA and MYC expression had a consistent increase in untreated SRSF2^mut^ cells and DNR-treated CTR cells compared to untreated CTR cells, along with DNR-induced BAX downregulation without changes in MCL1 or BCL-XL levels (Fig. 1D). Notably, while *SRSF2* mutation attenuated the antiproliferative and proapoptotic effects of HHT, Ara-C, AZA, and Dec, it did not reduce the sensitivity of the cells to Ven (Supplemental Figs. 3–7). Intriguingly, the mutation appeared to slightly increase Ven-induced apoptosis in these cells (Supplemental Fig. 7).

Kasumi-1 cells were engineered to express SRSF2^mut^ under a Dox-inducible promoter, with empty vector controls. Inducible expression was verified by WB and qPCR (Supplemental Fig. 8). Cytotoxicity analysis revealed reduced DNR sensitivity in SRSF2^mut^ cells (IC50 = 67.24 nM) compared with CTR cells (IC50 = 52.03 nM), although this difference was less significant than that in HEL-SRSF2^mut^ cells (Fig. 1E). Correspondingly, apoptosis assays revealed less DNR-induced apoptosis in mutant cells than in wild-type controls (Fig. 1F). WB analysis showed that PUMA and MYC expression were elevated in untreated SRSF2^mut^ cells compared with untreated CTR cells, whereas DNR-treated CTR cells exhibited specific upregulation of PUMA and BAX without alterations in the other examined proteins (Fig. 1G).

Unlike tumor cells with complex mutational backgrounds, IL-3-dependent BaF3 cells provide a genetically tractable model system for experimental analysis. Stable expression of SRSF2^mut^ was achieved in BaF3 cells via lentiviral transduction, with empty vector controls. Successful protein expression was confirmed by WB (Supplemental Fig. 9). Although SRSF2^mut^ cells were IL-3 dependent, their proliferation rates were significantly greater than those of CTR cells in IL-3-containing medium (Fig. 1H). Cytotoxicity assessment revealed reduced DNR sensitivity in SRSF2^mut^ cells (IC50 = 235.4 nM) compared with CTR cells (IC50 = 143.0 nM; Fig. 1I). This chemoresistance phenotype was corroborated by a reduced apoptotic response to DNR in mutant cells (Fig. 1J). Molecular characterization revealed baseline upregulation of antiapoptotic proteins (Mcl1, Bcl2) and Eif4e, concomitant with downregulation of Bax and CycD1 in untreated mutant cells. Notably, DNR treatment elevated both antiapoptotic (Mcl1, Bcl2) and proapoptotic (Bax) proteins, along with CycD1 (Fig. 1K).

### *SRSF2* mutation reduced DNR treatment efficacy in AML xenograft mouse models

**Fig. 2:**
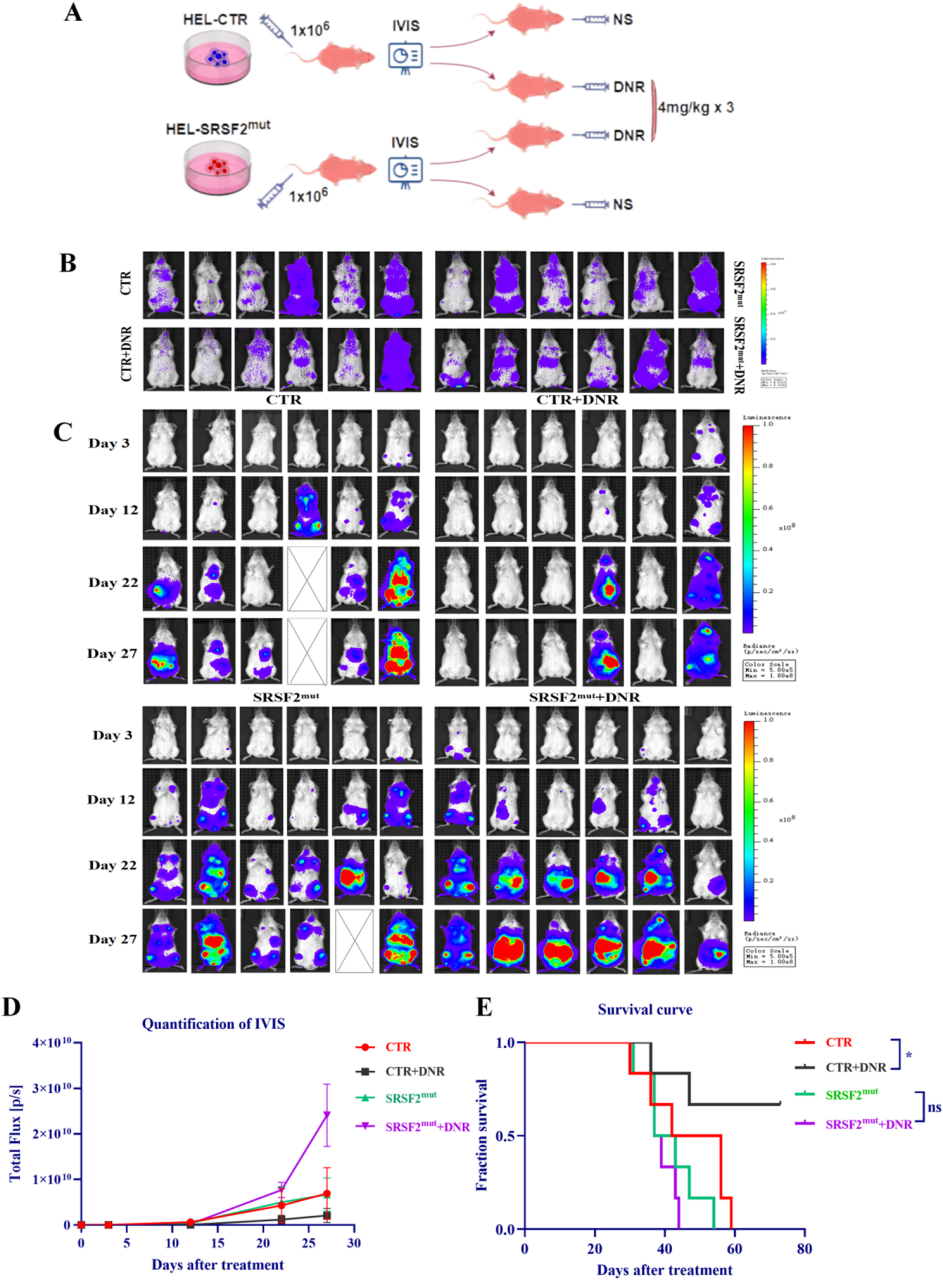
*SRSF2* mutation reduced survival in DNR-treated AML xenograft mice. (**A**) Schematic of the AML xenograft model establishment and treatment protocol (this schematic was generated by FigDraw). (**B**) IVIS imaging and experimental grouping on day 8 after tumor cell injection via the tail vein. (**C**) Longitudinal IVIS monitoring at days 3, 12, 22, and 27 after treatment initiation. (**D**) Quantitative assessment of the data shown in panel C. (**E**) Kaplan-Meier survival analysis. Abbreviations: DNR, daunorubicin hydrochloride; AML, acute myeloid leukemia; IVIS, *in vivo* imaging system

Mouse xenograft models of AML were established and subsequently stratified by tumor burden prior to DNR administration (Fig. 2A, B). With Day 1 defined as the first day of treatment initiation, tumor progression was serially evaluated via IVIS at Days 3, 12, 22, and 27. Bioluminescence quantification revealed divergent responses: while DNR-treated CTR mice presented attenuated tumor burden trajectories relative to those of untreated CTR mice, DNR-treated SRSF2^mut^ mice presented progressive tumor accumulation exceeding untreated SRSF2^mut^ levels (Fig. 2C, D). Consistent with these findings, survival analysis revealed that treatment benefited only CTR mice, with no therapeutic advantage conferred to SRSF2^mut^ mice, confirming *SRSF2* mutation-mediated DNR resistance *in vivo* (Fig. 2E).

**Fig. 3:**
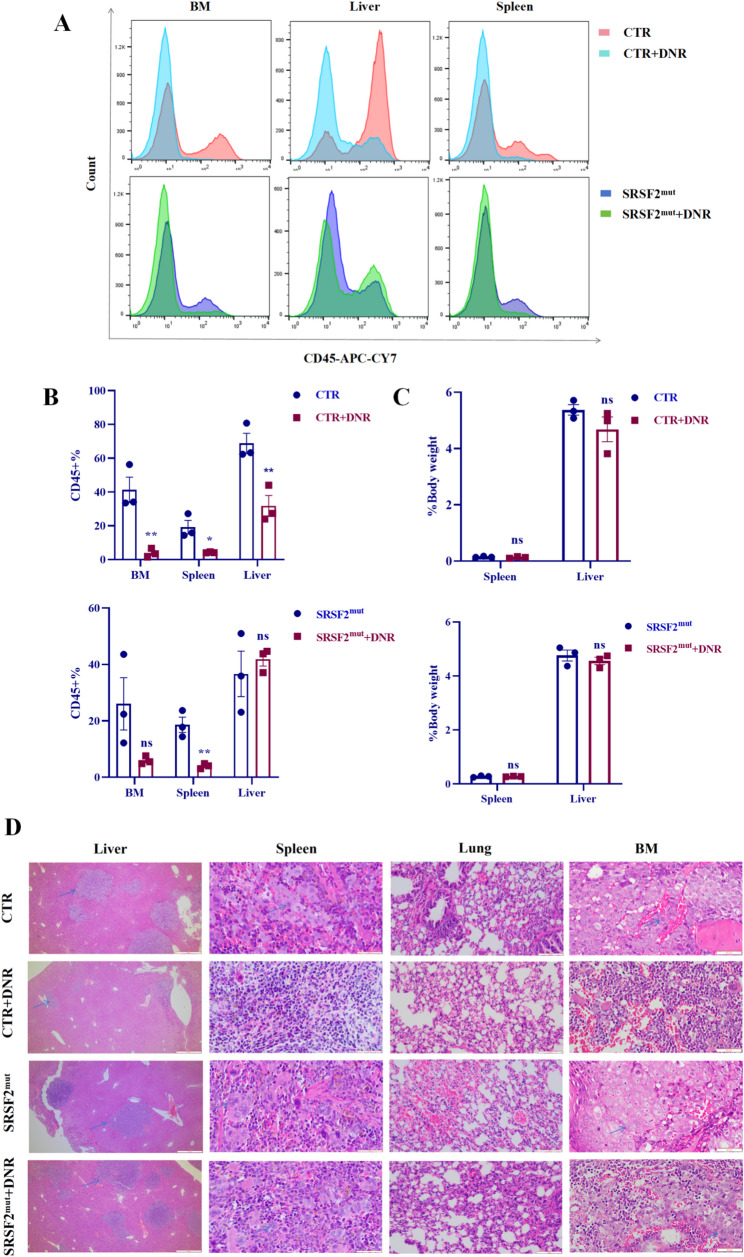
*SRSF2* mutation increased visceral leukemic infiltration in AML xenograft mice. (**A**) FCM analysis of CD45 + cell populations in the liver, spleen, and bone marrow. (**B**) Quantitative comparison of CD45 + cell infiltration across tissues. (**C**) Organ-to-body weight ratios of the liver and spleen. (**D**) Histopathological examination of multiple organs via H&E staining (Representative cancer cells are indicated by blue arrows). Abbreviations: AML, acute myeloid leukemia; FCM, flow cytometry; H&E, hematoxylin and eosin

FCM quantification of the CD45 + cell distribution revealed distinct organ-specific responses to DNR treatment (Fig. 3A, B). In CTR xenografts, DNR treatment significantly reduced leukemic infiltration in all evaluated organs (liver, spleen, and bone marrow). In contrast, SRSF2^mut^ xenografts displayed a selective response pattern, showing a reduction in only splenic infiltration while maintaining persistent hepatic involvement (with an increasing trend) and unaltered bone marrow infiltration posttreatment. Analysis of the organ-to-body weight ratios revealed no significant differences between the groups (Fig. 3C). H&E-stained sections of major organs (liver/spleen/bone marrow/lung) demonstrated differential therapeutic responses: DNR-treated CTR mice presented substantially decreased leukemic infiltration and attenuated tissue pathology, whereas SRSF2^mut^ mice maintained comparable levels of infiltration and tissue damage posttreatment (Fig. 3D).

### Investigating the molecular mechanisms of *SRSF2* mutation-associated drug resistance through RNA sequencing

Enrichment analysis of intersecting DEGs from comparisons between (i) untreated CTR cells and untreated SRSF2^mut^ cells and (ii) untreated CTR cells and DNR-treated CTR cells revealed mutation-related drug resistance mechanisms, as visualized via a Venn diagram (Supplemental Fig. 10A). Heatmap clustering of signature genes (red symbols in the Venn diagram) revealed both functional gene grouping (row clusters) and sample expression pattern similarity (column clusters) (Supplemental Fig. 10B). Multiplatform enrichment analysis revealed three key molecular pathways associated with *SRSF2* mutation-induced DNR resistance: the KEGG-annotated “transcriptional misregulation in cancer” pathway involving *ETV7*, *BIRC3*, *ITGB7*, and *JUP* (Supplemental Fig. 10C); the GO term “response to antineoplastic agent” encompassing *AQP1*, *GJB2*, *JUP*, and *PDGFB* (Supplemental Fig. 10D); and Reactome’s “*TNFR2* noncanonical *NF-κB* pathway” featuring *TNFRSF9*, *BIRC3*, and *RELB* (Supplemental Fig. 10E). The sequencing data were analyzed alongside the Beat AML dataset, resulting in the identification of seven DEGs, namely *N4BP3*, *PDGFB*, *GDF15*, *HES2*, *SLC4A1*, *DAPK2* and *GJB2* (Supplemental Fig. 11A). Enrichment analysis of DEGs between patients who were responsive and unresponsive to the treatment regimens containing DNR in the Beat AML dataset was performed (Supplemental Fig. 11B). The leukocyte migration biological process (containing *IL1B*, *THBS1* and *PDGFB* genes) overlapped with the results of the enrichment analysis of DEGs between the CTR and SRSF2^mut^ groups.

**Fig. 4:**
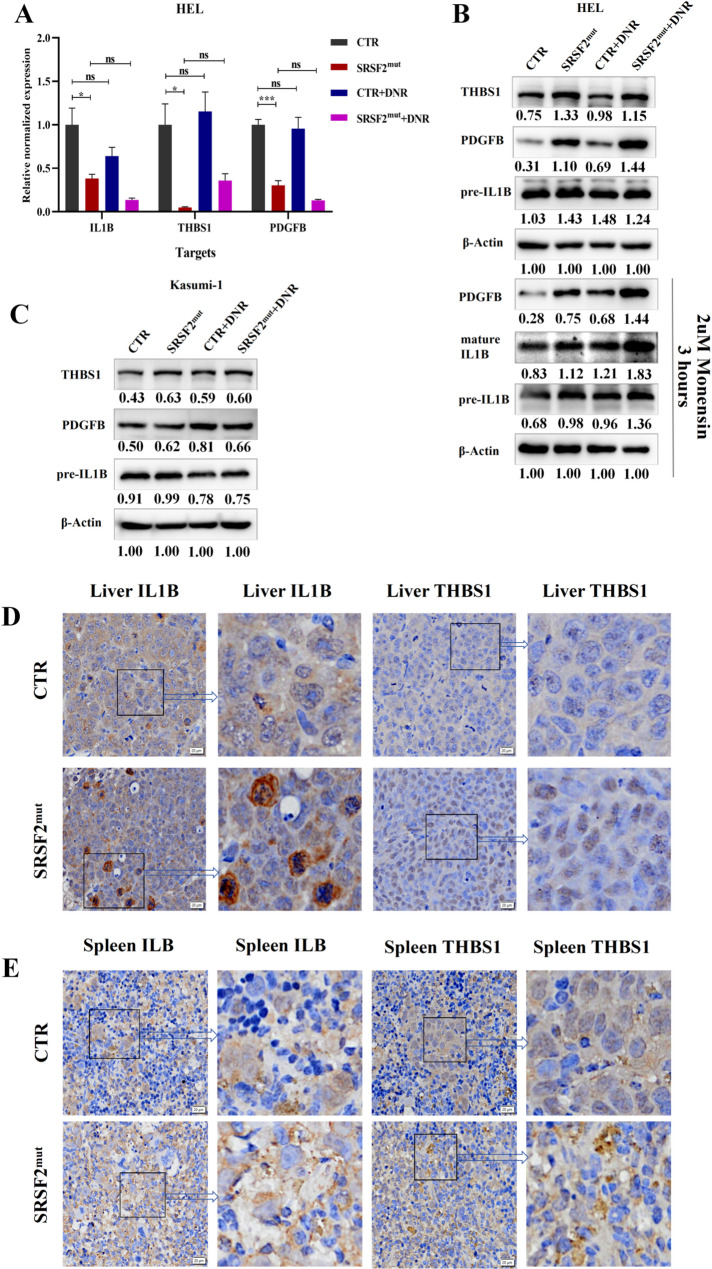
The transcriptional activity of the leukocyte migration pathway was quantified, and the protein expression levels of the genes were detected by WB and IHC. (**A**) Transcript levels of genes within the leukocyte migration pathway. (**B**) Upper panel: WB analysis of protein expression in HEL cells treated for 16 h with 1× PBS or DNR; lower panel: additional 3-hour treatment with monensin (2 µM final concentration) followed by WB. (**C**) WB analysis of protein levels in Kasumi-1 cells following 16 h of exposure to 1× PBS or DNR. (**D**, **E**) IHC detection of THBS1 and IL1B protein expression and distribution in liver and spleen tissues from AML xenograft model mice. Abbreviations: WB, western blotting; IHC, immunohistochemistry; PBS, phosphate-buffered saline; DNR, daunorubicin hydrochloride; AML, acute myeloid leukemia

qPCR was used to examine differences in the transcript levels of relevant genes or signaling pathways between different groups. The results revealed that the *ETV7* gene was upregulated in the SRSF2^mut^ cells, whereas the *ITGB7*, *RELB*, *TNFRSF9*, *IL1B*, *THBS1*, *PDGFB*, *JUP* and *GJB2* genes were downregulated compared with those in the CTR cells. DNR treatment caused the upregulation of *ETV7* and *GJB2* but the downregulation of *BIRC3* in the CTR cells, with no significant differences in the transcript levels of other genes (Supplemental Fig. 10F-H). As these genes were the focus of subsequent investigations, the expression of genes involved in the leukocyte migration biological process (*IL1B*, *THBS1*, *PDGFB*) was characterized separately.(Fig. 4A).

The mRNA level is an important predictor of protein synthesis, but the latter is regulated at multiple levels, such as posttranscriptional regulation [33], translation efficiency, and posttranslational modification [34]. Therefore, we detected changes in the protein levels of the aforementioned genes (Fig. 4B, C; Supplemental Fig. 12). The results revealed that *SRSF2* mutation led to the upregulation of THBS1, PDGFB or IL1B in cells, whereas JUP, AQP1, GJB2, BIRC3 and RelB were downregulated. DNR treatment resulted in the upregulation of PDGFB, mature IL1B, THBS1 or AQP1 in the CTR cells, whereas BIRC3 and RelB were slightly downregulated. IHC analysis of liver and spleen tissues from the xenograft AML mouse model provided further evidence that *SRSF2* mutation could upregulate the expression of THBS1 and IL1B *in vivo* (Fig. 4D, E).

### *SRSF2* mutation altered *THBS1* mRNA stability and translation efficiency

**Fig. 5:**
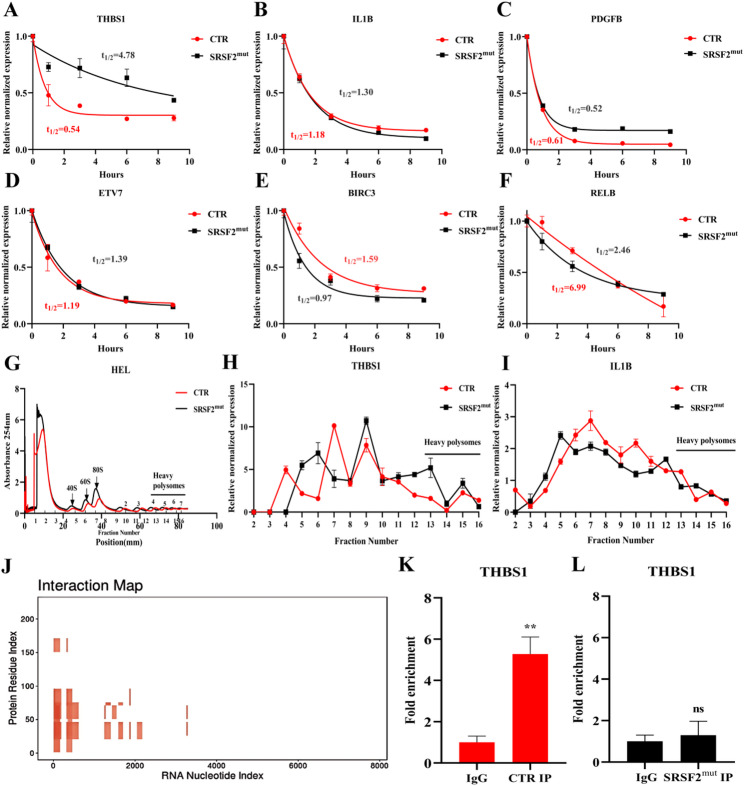
*SRSF2* mutation significantly altered *THBS1* mRNA stability and translation efficiency. (**A**-**F**) Effects of *SRSF2* mutation on the mRNA half-life of related genes in HEL cells were assessed through RNA stability assays. (**G**) Polysome profile analysis revealed the impact of *SRSF2* mutation on the overall translational landscape in HEL cells. (**H**) Quantitative distribution of *THBS1* mRNA in ribosomal fractions. (**I**) Quantitative distribution of *IL1B* mRNA in ribosomal fractions. (**J**) In silico prediction of SRSF2-*THBS1* transcript interactions via catRAPID omics v2.1. (**K**) RIP-qPCR analysis of *THBS1* in CTR cells. (**L**) RIP-qPCR analysis of *THBS1* in SRSF2^mut^ cells. Abbreviations: RIP, RNA immunoprecipitation; qPCR, real-time quantitative polymerase chain reaction

We first performed mRNA stability assays and observed that *SRSF2* mutation notably extended the half-life of *THBS1* mRNA in cells (Fig. 5A-F). mRNA stability and translation efficiency are interrelated [35]; therefore, we next assessed the translation efficiency of mRNAs in cells via polysome profiling. This analysis revealed that *SRSF2* mutation had no significant effect on overall cellular translation (Fig. 5G). The distribution of *THBS1* or *IL1B* mRNA across different ribosome fractions was assessed via qPCR. Compared with that in the CTR group, the relative expression of *THBS1* mRNA in the SRSF2^mut^ group was greater in the heavy polysome fraction (containing more than three ribosomes), which represents mRNAs with high translation efficiency (Fig. 5H). The relative expression of *IL1B* mRNA in the SRSF2^mut^ cells was lower than that in the CTR cells in the polysome fraction containing four ribosomes but higher in the fraction containing five ribosomes than in the CTR cells; however, the translation efficiency of mRNA was greater in the polysome fraction containing five ribosomes (Fig. 5I).

We used catRAPID omics v2.1 to predict the binding potential between SRSF2 protein and RNAs of interest, and identified that SRSF2 protein could bind to *THBS1* transcripts (Fig. 5J). The RIP assays revealed an interaction between SRSF2 protein and *THBS1* mRNA in CTR cells, but this interaction was absent in SRSF2^mut^ cells (Fig. 5K, L). Since SRSF2 is a splicing factor that interacts with *THBS1* mRNA, we further analyzed the alternative splicing results from the RNA-seq data and found that *THBS1* exhibited two types of alternative splicing: intron retention and exon skipping. However, *SRSF2* mutation did not affect splicing events of *THBS1* (Supplemental Fig. 13).

### *SRSF2* mutation altered *ETV7* alternative splicing patterns

Combining the results of alternative splicing analysis from the RNA-seq data with those of qPCR validation via the use of specifically designed primers, we found that *SRSF2* mutation affected alternative splicing of *ETV7* and increased its expression level (Supplemental Fig. 14). Additionally, DNR treatment also elevated the expression of *ETV7* in cells (Supplemental Fig. 14). Both *SRSF2* mutation and DNR treatment upregulated the expression of *ETV7* in leukemic cells, suggesting that *ETV7* might play a role in the resistance mechanism of SRSF2^mut^ cells to DNR treatment.

### *SRSF2* mutation enhanced mitochondrial oxidative phosphorylation

Changes in cellular metabolism, including glycolysis, mitochondrial function, and fatty acid metabolism, are important factors leading to a reduced drug response or resistance development [36]. We used a mitochondrial stress test to evaluate mitochondrial function and the mitochondrial response under various stress conditions. Compared with CTR cells, SRSF2^mut^ cells presented increased spare respiratory capacity (Supplemental Fig. 15A). We further measured the glycolytic rate in cells and found that, compared with CTR cells, SRSF2^mut^ cells presented a slight decrease in basal glycolysis, whereas compensatory glycolysis remained unchanged (Supplemental Fig. 15B).

### Differential expression of *ETV7*, *IL1B*, *THBS1*, and *PDGFB* in normal vs. AML tissues

Analysis of TCGA and GTEx data through the GEPIA platform revealed distinct expression profiles between normal and AML samples: *ETV7* presented comparable expression levels between groups, whereas *IL1B* and *THBS1* were significantly upregulated in AML samples (Supplemental Fig. 16A-C). In contrast, *PDGFB* was expressed at higher levels in normal tissues than in AML samples (Supplemental Fig. 16D).

### Prognostic value of altered *ETV7*, *PDGFB*, and *THBS1* expression in AML patients

Analysis of multiple datasets (GSE12417, GSE106291, GSE165656, Beat AML, and TCGA) via R software revealed significant associations between gene expression patterns and patient outcomes. High-*ETV7*-expressing patients had poorer survival than low-*ETV7*-expressing patients (Supplemental Fig. 17A). Although *PDGFB* expression did not differ prognostically in the Beat AML and GSE12417 cohorts, high expression correlated with worse outcomes in the other three datasets (Supplemental Fig. 17B). Notably, although *PDGFB* expression was higher in normal tissues than in AML samples, its relatively increased expression in AML patients correlated with adverse prognosis, suggesting context-dependent roles, potentially maintaining normal cellular functions in healthy tissues while promoting proliferation or apoptosis resistance in AML. Patients with high *THBS1* expression also had inferior survival (Supplemental Fig. 17C). *SRSF2* mutation was associated with elevated protein levels of the *ETV7*, *PDGFB*, and *THBS1* genes in cells. Analysis of public databases revealed that increased expression levels of these genes were associated with a poorer prognosis in AML patients, suggesting that the poor prognosis associated with *SRSF2* mutation might be related to the altered expression patterns of these genes.

### Synergistic antileukemic activity of DNR and CP-673451 in SRSF2^mut^ AML cells

**Fig. 6:**
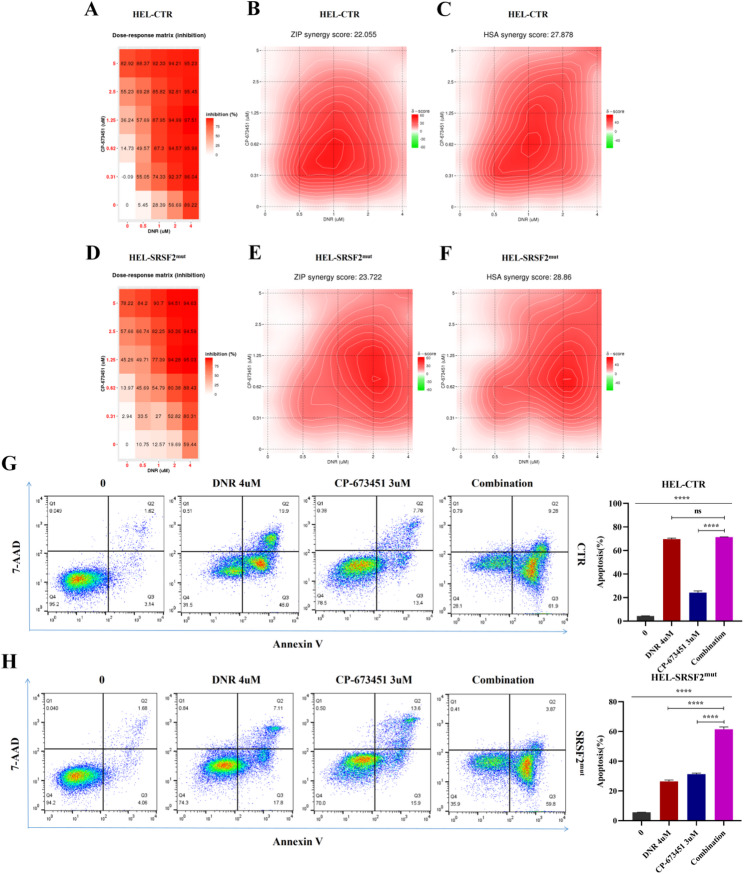
Synergistic cytotoxicity of CP-673451 and DNR in HEL cells. (**A**) Dose matrix for the CTR group. (**B**) ZIP synergy model (CTR). (**C**) HSA synergy model (CTR). (**D**) Dose matrix for the SRSF2^mut^ group. (**E**) ZIP synergy model (SRSF2^mut^). (**F**) HSA synergy model (SRSF2^mut^). (**G**) FCM detection of proapoptotic effects in CTR cells treated with DNR, CP-673451 or their combination. (**H**) FCM detection of proapoptotic effects in SRSF2^mut^ cells treated with DNR, CP-673451 or their combination. Note that in the synergy heatmaps, a gradient from red to green is interpreted as a transition from synergy (red, higher scores) to antagonism (green, lower scores). Abbreviations: DNR, daunorubicin hydrochloride; CTR, control; FCM, flow cytometry; ZIP, zero interaction potency; HSA, highest single agent

**Fig. 7:**
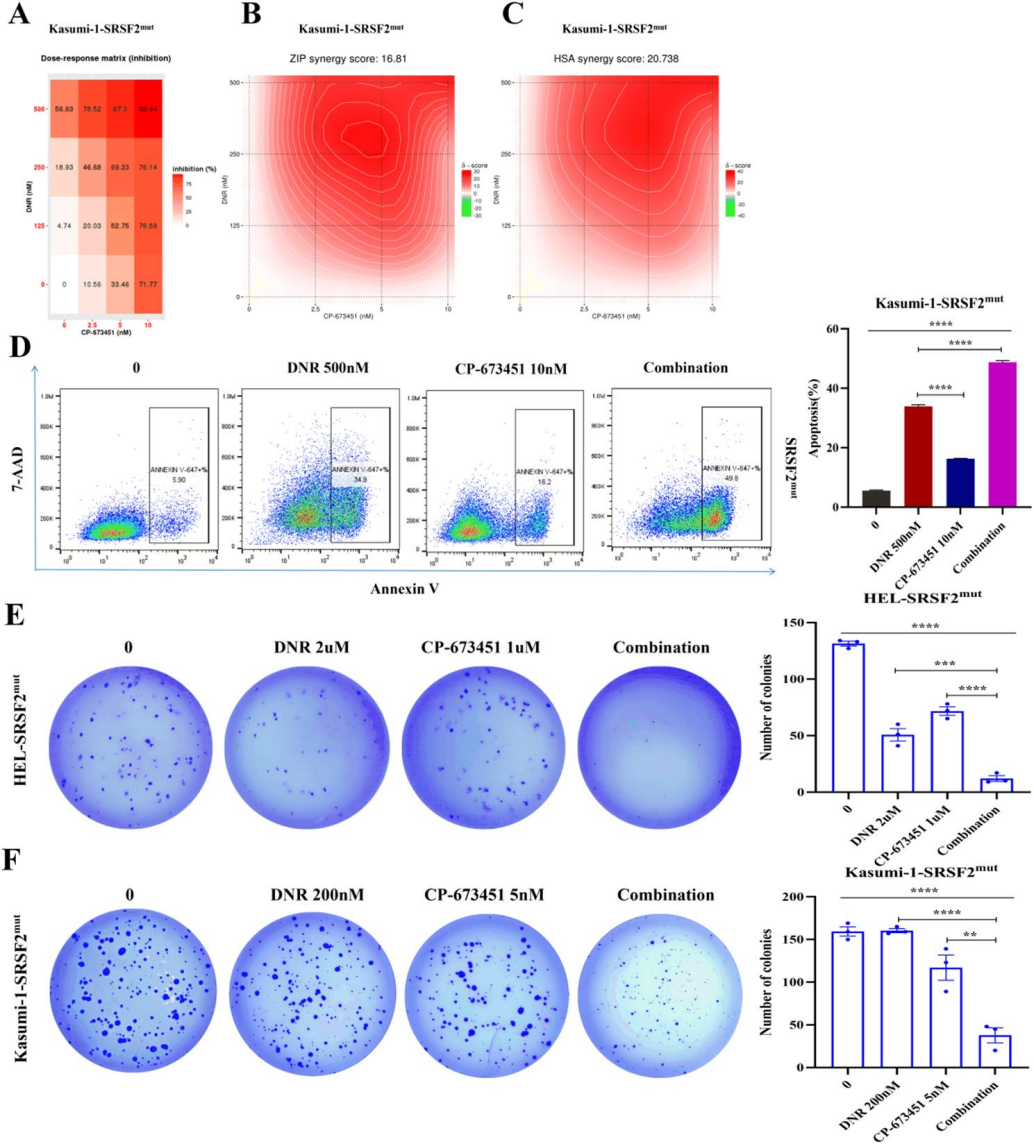
Synergistic cytotoxicity of CP-673451 and DNR in SRSF2^mut^ Kasumi-1 cells. (**A**) Drug dose matrix. (**B**) ZIP synergy model. (**C**) HSA synergy model. (**D**) Proapoptotic effects of DNR, CP-673451 or their combination detected by FCM. (**E**) Colony formation of SRSF2^mut^ HEL cells treated with CP-673451, DNR, or their combination (left: representative images; right: quantitative analysis). (**F**) Colony formation of SRSF2^mut^ Kasumi-1 cells treated with CP-673451, DNR, or their combination (left: representative images; right: quantitative analysis). Note that in the synergy heatmaps, a gradient from red to green is interpreted as a transition from synergy (red, higher scores) to antagonism (green, lower scores). Abbreviations: DNR, daunorubicin hydrochloride; FCM, flow cytometry; ZIP, zero interaction potency; HSA, highest single agent

Both *SRSF2* mutation and DNR treatment upregulated PDGFB expression, which activated receptor-mediated signaling to promote leukemic cell survival and proliferation [37]. We investigated the therapeutic potential of combining DNR with CP-673451, a PDGFR inhibitor, with a focus on SRSF2^mut^ cells. MTT assays demonstrated that CP-673451 was more potent in SRSF2^mut^ cells (IC50 = 1.69 µM) than in CTR cells (IC50 = 1.99 µM), consistent with mutation-associated PDGFB upregulation. Synergy analysis revealed stronger combined effects in the mutants (ZIP:23.7; HSA:28.9) than in the controls (ZIP:22.1; HSA:27.9) (Fig. 6A-F). FCM revealed that CP-673451 increased DNR-induced apoptosis in SRSF2^mut^ cells (from 26.4% to 61.5%) but had a minimal effect on CTR cells (from 69.7% to 71.3%) (Fig. 6G, H). These findings were validated in Kasumi-1 cells, where the combination had significant synergistic effects (ZIP: 16.8; HSA: 20.7) and increased apoptosis from 33.9% to 48.8% (Fig. 7A-D). These results demonstrated that PDGFB inhibition effectively reversed *SRSF2* mutation-mediated DNR resistance, with the combination showing mutation-selective synergy through increased apoptosis. The colony-forming ability of SRSF2^mut^ cells was quantitatively evaluated via soft agar assays following exposure to CP-673451, DNR, or their combination. Compared with the individual drug treatments, the dual treatment significantly suppressed both colony formation frequency and dimensions, demonstrating substantially compromised long-term proliferative potential (Fig. 7E, F). These data support PDGFR blockade as a strategy to overcome chemoresistance in SRSF2^mut^ AML.

## Discussions

*SRSF2* has diverse biological functions, including the regulation of pre-mRNA splicing, the modulation of RNA stability, transcriptional control, and translational regulation, all of which contribute to maintaining normal physiological processes and cellular functions [38]. *SRSF2* mutations have been demonstrated to actively contribute to the initiation and progression of AML [39]. Our clinical sequencing identified nine mutation sites, predominantly clustered between the RRM and RS domains of SRSF2, consistent with known mutational hotspots [40]. While the initial survival curves showed no difference, the exclusion of HSCT recipients resulted in worse outcomes for patients with *SRSF2* mutations. The role of *SRSF2* extends beyond prognosis to therapy resistance, with documented impacts on the sorafenib response in hepatocellular carcinoma [41] and on cisplatin sensitivity in ovarian cancer [42, 43]. Although *SRSF2* is consistently linked to adverse outcomes in AML, the mechanistic basis of *SRSF2*-mediated resistance remains undefined. In engineered SRSF2^mut^ models, conventional AML therapies (DNR, HHT, Ara-C, AZA, and Dec) elicited attenuated responses, most markedly for DNR. In contrast, Ven efficacy was maintained, even showing slightly enhanced pro-apoptotic effects. These preclinical findings mirror clinical reports where splicing factor-mutated AML patients presented outcomes comparable to those of their wild-type counterparts when treated with hypomethylating agent/Ven regimens, particularly those with *SRSF2*/*IDH2* comutations [44]. *SRSF2* mutations induced significant alterations in the expression of critical cell cycle and apoptosis regulators, most notably resulting in elevated PUMA and MYC levels. At the mechanistic level, SRSF2 participates in the regulation of p53 posttranslational modifications, where its functional deficiency causes aberrant p53 hyperphosphorylation, as demonstrated in mouse embryonic fibroblast models [45]. Since p53 serves as a direct transcriptional regulator of PUMA [46], this established pathway provides a molecular basis for the observed effects of *SRSF2* mutations on PUMA expression patterns.

*In vitro* drug screening revealed that SRSF2^mut^ cells exhibited significantly reduced sensitivity to DNR-induced cytotoxicity. To validate these findings *in vivo*, we assessed the effect of *SRSF2* mutation on DNR treatment efficacy in xenograft AML mouse models. SRSF2^mut^ cells develop DNR resistance and may acquire enhanced aggressiveness under treatment pressure, potentially through drug resistance mechanisms such as efflux pump upregulation. Moreover, subtherapeutic DNR dosing in SRSF2^mut^ mice appeared to exacerbate tumor progression, possibly due to both insufficient leukemia control and drug-related systemic toxicity. Survival analysis revealed that while DNR prolonged survival in the CTR group, it provided no benefit to SRSF2^mut^ mice, suggesting that the mutation compromises DNR efficacy by altering drug responsiveness. FCM and H&E analyses of organ infiltration further confirmed mutation-associated therapeutic resistance.

To elucidate the pathways underlying *SRSF2* mutation-associated DNR resistance, we integrated RNA-seq data from cultured cells with DGE analysis of DNR responders versus nonresponders from the Beat AML dataset. qPCR assays confirmed that the transcriptional changes were consistent with the sequencing results, and the corresponding protein level alterations were examined. WB analysis revealed that *SRSF2* mutation-mediated upregulation of THBS1, PDGFB, and IL1B occurred alongside the downregulation of JUP, AQP1, GJB2, BIRC3, and RelB. While DNR treatment upregulated PDGFB, mature IL1B, THBS1, and AQP1 in CTR cells, it only moderately reduced BIRC3 and RelB expression. *In vivo* validation via IHC confirmed *SRSF2* mutation-driven THBS1 and IL1B overexpression in xenograft liver/spleen tissues. THBS1, which functions through CD47/CD36 interactions, mediates platelet aggregation, angiogenesis, and immune modulation [47, 48], whereas in malignancies, it fosters immunosuppressive microenvironments that promote metastasis (colorectal cancer) [49] and activate PI3K/AKT-driven proliferation (oral squamous cell carcinoma) [50]. PDGFB orchestrates tumor angiogenesis and progression [37], synergizing with TGF-β to enhance immune evasion [51], with its dimer PDGF-BB promoting cholangiocarcinoma metastasis [52] and gliomagenesis [53]. Proinflammatory IL1B accelerates AML progression via the induction of tousled-like kinases/antisilencing function-1B [54] while conferring chemoresistance through the PGE2/Cox-2/β-catenin pathways [55]. Notably, PDGFB and IL1B are cross-regulated, with PDGFB-stimulated glioblastoma cells inducing macrophage IL1B production, which subsequently activates NF-κB [56]. The downregulated NF-κB component RelB regulates immune responses and survival [57], contributing to PDL1-mediated immune escape in prostate cancer [58] and GPX4-dependent tamoxifen resistance in breast cancer [59]. Similarly, BIRC3 depletion, through its dual roles in apoptosis inhibition and NF-κB-mediated immune regulation [60], drives chemoresistance in hematologic malignancies such as fludarabine-refractory CLL [61]. Notably, our protein-level validation results revealed that the regulation of these key molecules by SRSF2 mutation and DNR treatment exhibited significant cell line specificity. The most pronounced differences were observed in PDGFB and BIRC3. In HEL cells, the *SRSF2* mutation-induced upregulation of PDGFB protein was more prominent, and DNR treatment further strongly enhanced its expression; this robust PDGFB survival signaling axis may have constituted a core mechanism for HEL cells to resist DNR stress. In Kasumi-1 cells, the basal protein level of BIRC3 was markedly downregulated by *SRSF2* mutation. As a key regulator in the TNFR2 noncanonical NF-κB pathway, the profound downregulation of BIRC3 may have led to aberrant activation of this pathway, ultimately contributing to the resistant phenotype. This differential regulatory pattern of PDGFB and BIRC3 across different cellular contexts suggested that the *SRSF2* mutation drove the decrease in DNR sensitivity through heterogeneous molecular pathways.

Our comprehensive analysis combining qPCR, WB, and IHC data revealed discordant expression patterns between the mRNA and protein levels of the leukocyte migration-related genes *IL1B*, *THBS1*, and *PDGFB* in SRSF2^mut^ cells, with decreased mRNA but increased protein expression. Previous studies have indicated that *SRSF2* mutations influence mRNA stability, as exemplified by their association with *EZH2* splicing inactivation through retention of a toxic cassette exon containing a premature termination codon that induces nonsense-mediated decay [62]. mRNA stability assays revealed that *SRSF2* mutation markedly extended *THBS1* mRNA half-life, although no significant differences in the levels of *IL1B* and *PDGFB* were detected between the groups. Considering the interdependent nature of mRNA stability and translation efficiency [35], where unstable mRNAs face increased nuclease degradation with diminished translation potential and ribosomal stalling can promote mRNA decay [63], we employed polysome profiling to evaluate translational efficiency. qPCR analysis of ribosomal fractions revealed increased translational efficiency of *THBS1* mRNA in SRSF2^mut^ cells, whereas *IL1B* mRNA presented increased relative expression in five-ribosome fractions, implying increased overall translation efficiency in mutant cells. *SRSF2* mutations have differential effects on RNA motif binding affinity, resulting in enhanced binding to UCCAG and UGCAG sequences but weakened interactions with UGGAG sites relative to those of the wild-type protein [64]. The wild-type SRSF2 protein preferentially binds to 5-methylcytosine (m5C)-modified mRNAs, a capacity that is compromised by mutations. Importantly, decreased m5C modification through approaches such as *NSUN2* knockdown similarly reduces *SRSF2* mRNA binding affinity, mirroring the splicing alterations observed with SRSF2 depletion [65]. To investigate potential SRSF2-RNA interactions, we initially employed catRAPID omics v2.1 to predict binding probabilities, which identified possible associations between SRSF2 and *THBS1* transcripts. RIP-qPCR validation subsequently confirmed the SRSF2-*THBS1* mRNA interaction in CTR cells, an interaction that was absent in SRSF2^mut^ cells, demonstrating mutation-mediated binding disruption. Despite SRSF2’s established role as a splicing factor that interacts with *THBS1* mRNA, examination of RNA-seq splicing profiles revealed that *SRSF2* mutation did not substantially influence *THBS1* alternative splicing patterns, including intron retention and exon skipping events.

Through integrated analysis of RNA-seq data on alternative splicing and validation via specific-primer qPCR, we demonstrated that *SRSF2* mutation altered *ETV7* alternative splicing and increased its expression level. Notably, DNR treatment also elevated *ETV7* expression in cells. *ETV7* is a transcription factor with multiple biological functions, including the regulation of immune responses and cell proliferation/differentiation [66]. Its overexpression has been detected in various cancers where it promotes tumor progression; for example, upregulated *ETV7* reduces apoptosis while enhancing proliferation, migration, and cell cycle progression in colorectal cancer cells [67]. In breast cancer cells, elevated *ETV7* expression increases stemness and proliferative activity, confers resistance to chemotherapy and radiotherapy, and simultaneously suppresses inflammatory responses by inhibiting the *TNFR1*/*NF-κB* pathway [68]. The concurrent upregulation of *ETV7* by both *SRSF2* mutation and DNR treatment suggested its potential involvement in mediating drug resistance in SRSF2^mut^ cells during DNR therapy.

Metabolic reprogramming is a critical determinant of therapeutic resistance. Specifically, CD96-mediated augmentation of mitochondrial fatty acid β-oxidation confers chemoresistance in breast cancer stem cells [36, 69], while AML cells maintain survival, stemness, and drug tolerance through enhanced oxidative phosphorylation and fatty acid oxidation dependence [70]. *SRSF2* mutations potentiated mitochondrial oxidative phosphorylation to meet increased energy demands during cellular stress, thereby promoting survival. Notably, Ara-C-resistant AML cells exhibit mitochondrial expansion with sustained polarization and hyperactivity, mirroring their elevated oxidative phosphorylation capacity and *in vivo* chemoresistance [71]. Mechanistically, CD39 upregulation in resistant cells triggers cAMP-dependent mitochondrial stress responses that increase biogenesis and functional capacity, ultimately driving Ara-C resistance [72]. Conversely, disrupting mitochondrial transfer from stromal cells or inhibiting oxidative phosphorylation sensitizes AML cells to Ara-C [73]. These findings collectively suggested that *SRSF2* mutations may confer chemoresistance through enhanced mitochondrial function.

**Fig. 8:**
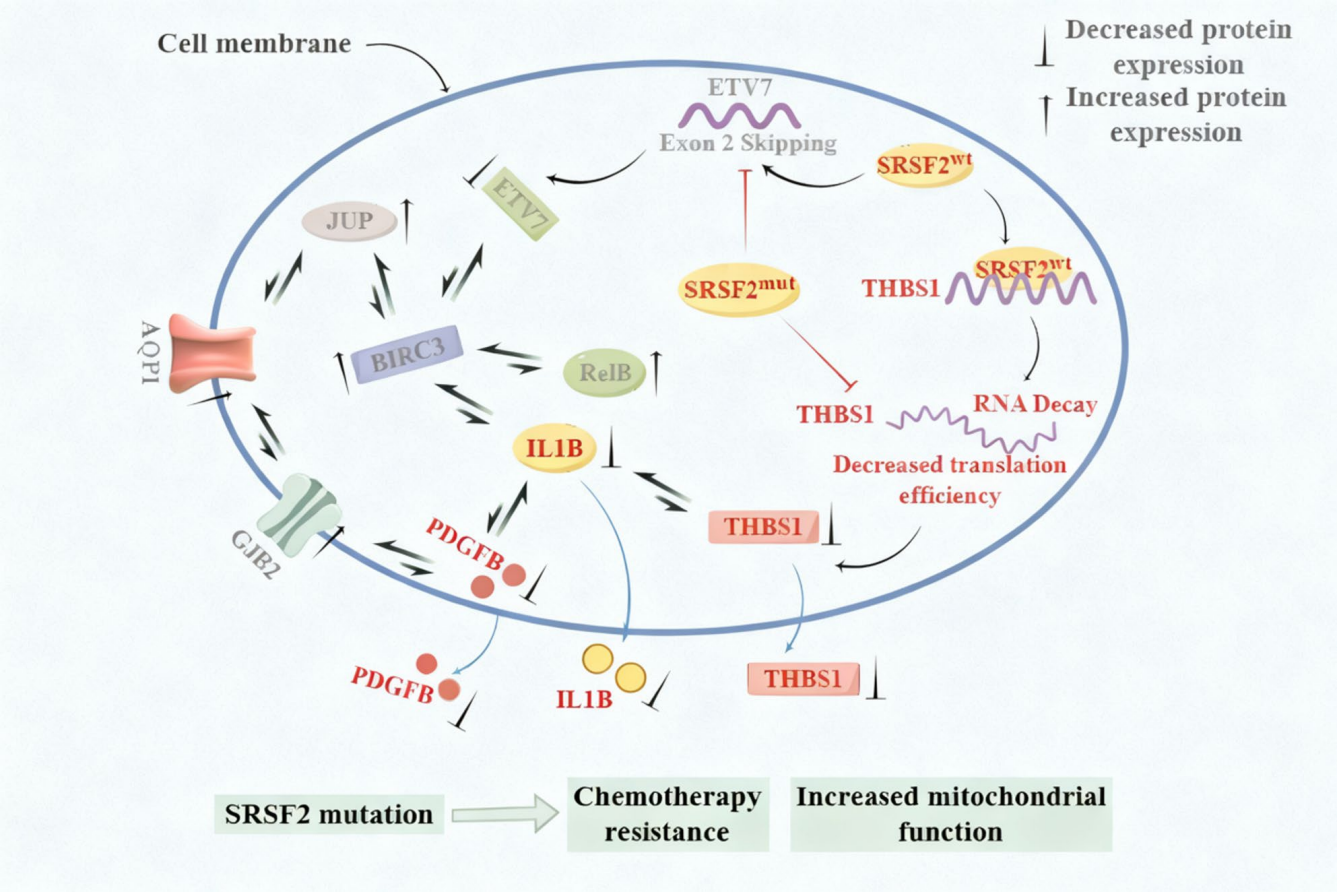
*SRSF2* mutation-altered target gene expression and pathway activation. The leukocyte migration pathway (THBS1, PDGFB, IL1B, in red) constitutes the core mechanism. Alterations in *ETV7* splicing and its associated effects (in gray) represent potential contributing pathways.This schematic was generated by FigDraw

RNA-binding proteins regulate gene expression by controlling mRNA modification, maturation, stability, and translation, thereby maintaining organismal homeostasis [74]. As both an RNA-binding protein and a splicing factor, SRSF2 modulates alternative splicing, stability, and translation of mRNAs. For example, *SRSF2* mutations induce nonsense-mediated decay of *EZH2* mRNA [62] while increasing both the mRNA stability and protein abundance of *PINK1*, a mitophagy activator [75]. *SRSF2* mutation might impact cellular biological functions through the following molecular mechanisms (Fig. 8). The interaction between SRSF2 protein and *THBS1* mRNA might decrease the stability and translation efficiency of the latter, maintaining THBS1 protein expression at a low level. Consequently, PDGFB and IL1B proteins are maintained at low levels. In addition, the SRSF2 protein regulates ETV7 expression by affecting its alternative splicing, thereby maintaining it at a relatively low level. Previous studies have shown that the expression levels of ETV7, BIRC3, RelB, JUP and IL1B are interrelated. When the interaction between SRSF2 protein and *THBS1* mRNA is weakened or lost due to *SRSF2* mutation, it might prolong the half-life of *THBS1* mRNA, increase its translation efficiency, and result in a relative increase in THBS1 protein expression, which further elevates the protein levels of PDGFB and IL1B. Moreover, *SRSF2* mutation alters the splicing pattern of *ETV7*, resulting in a relative increase in its expression and subsequently affecting the expression of BIRC3, RelB, JUP, or IL1B. Collectively, these findings delineate potential mechanisms by which *SRSF2* mutation influences the cellular phenotype. Among them, the leukocyte migration pathway (THBS1, IL1B, PDGFB) constitutes the central mechanism under investigation, whereas the other pathways are potential contributors to the overall phenotype.

PDGFB activates its cognate receptor, thereby initiating or pathologically enhancing intracellular signaling pathways that promote cancer cell survival, proliferation, and migration [37]. CP-673451 inhibits the action of PDGFB by competitively binding to PDGFR. *SRSF2* mutation slightly increased the sensitivity of cells to CP-673451, and when the latter was combined with DNR, it synergistically inhibited the proliferation and colony-forming abilities of SRSF2^mut^ cells while synergistically promoting their apoptosis. This finding indirectly implied that the inhibition of PDGFB function enhanced the sensitivity of cells to DNR, suggesting that PDGFB played a role in the resistance mechanism against DNR.

## Conclusions

*SRSF2* mutations confer poor prognosis in AML patients. The antiproliferative and proapoptotic effects of DNR, HHT, Ara-C, AZA, and Dec were all attenuated in SRSF2^mut^ cells, with DNR showing the most pronounced reduction in efficacy, whereas sensitivity to Ven remained unaffected. DNR treatment improved survival outcomes in CTR mice but did not improve survival in SRSF2^mut^ mice. Mechanistically, *SRSF2* mutation dysregulated THBS1/IL1B/PDGFB or ETV7/BIRC3/RelB signaling networks, which functionally intersected with DNR mechanisms of action, or alternatively impaired cellular responsiveness to these signals, collectively diminishing DNR sensitivity. Notably, *SRSF2* mutation slightly enhanced the sensitivity of cells to CP-673451, which exhibited synergistic effects with DNR in terms of suppressing proliferation and clonogenic potential, and promoting apoptosis in SRSF2^mut^ cells. This discovery has significant clinical and research implications. The combination of the two drugs could not only increase the efficacy of DNR but also reduce its therapeutic dose, thereby alleviating side effects and minimizing toxicity to normal cells. This approach might improve the prognosis for this group of patients.

## Supplementary Information

Supplementary Material 1.

Supplementary Material 2.

## Data Availability

All data relevant to the study are included in the article or uploaded as supplementary information.

## Abbreviations

AML: Acute myeloid leukemia
HSCT: Hematopoietic stem cell transplantation
AA: Antimycin A
Ara-C: Cytarabine
AZA: Azacitidine
BSD: Blasticidin
CTR: Control
cDNA: Complementary DNA
CHX: Cycloheximide
DNR: Daunorubicin hydrochloride
Dec: Decitabine
Dox: Doxycycline hyclate
FCM: Flow cytometry
HHT: Homoharringtonine
IVIS: *In vivo* imaging system
H&E: Hematoxylin and eosin
IHC: Immunohistochemistry
qPCR: Real-time quantitative polymerase chain reaction
RIP: RNA immunoprecipitation
WB: Western blotting
7-AAD: 7-aminoactinomycin D
Rot: Rotenone
Ven: Venetoclax
FCCP: Carbonyl cyanide-4 (trifluoromethox) phenylhydrazone
2-DG: 2-deoxy-D-glucose
OCR: Oxygen consumption rate
ECAR: Extracellular acidification rate
PBS: Phosphate-buffered saline
DEGs: Differentially expressed genes
KEGG: Kyoto Encyclopedia of Genes and Genomes
ZIP: Zero interaction potency
HSA: Highest single agent
SRSF2: Serine/arginine-rich splicing factor 2
PDGFB: Platelet-derived growth factor subunit B
PDGFR: Platelet-derived growth factor receptor
THBS1: Thrombospondin-1
ETV7: ETS variant transcription factor 7
BIRC3: Baculoviral IAP repeat containing 3
ITGB7: Integrin subunit beta 7
JUP: Junction plakoglobin
AQP1: Aquaporin 1
GJB2: Gap junction protein beta 2
TNFRSF9: TNF receptor superfamily member 9
RELB: RELB proto-oncogene, NF-kB subunit
IL1B: Interleukin 1 beta
