# Supplementary material for: SRSF2 mutations drive daunorubicin resistance in acute myeloid leukemia via THBS1 stabilization
Source: J Exp Clin Cancer Res. 2026 Jan 30;45:64. doi: 10.1186/s13046-026-03649-y (PMC12973616; doi:10.1186/s13046-026-03649-y)
Supplement: Supplementary file 1 — Supplementary Material 1. [file 13046_2026_3649_MOESM1_ESM.docx]

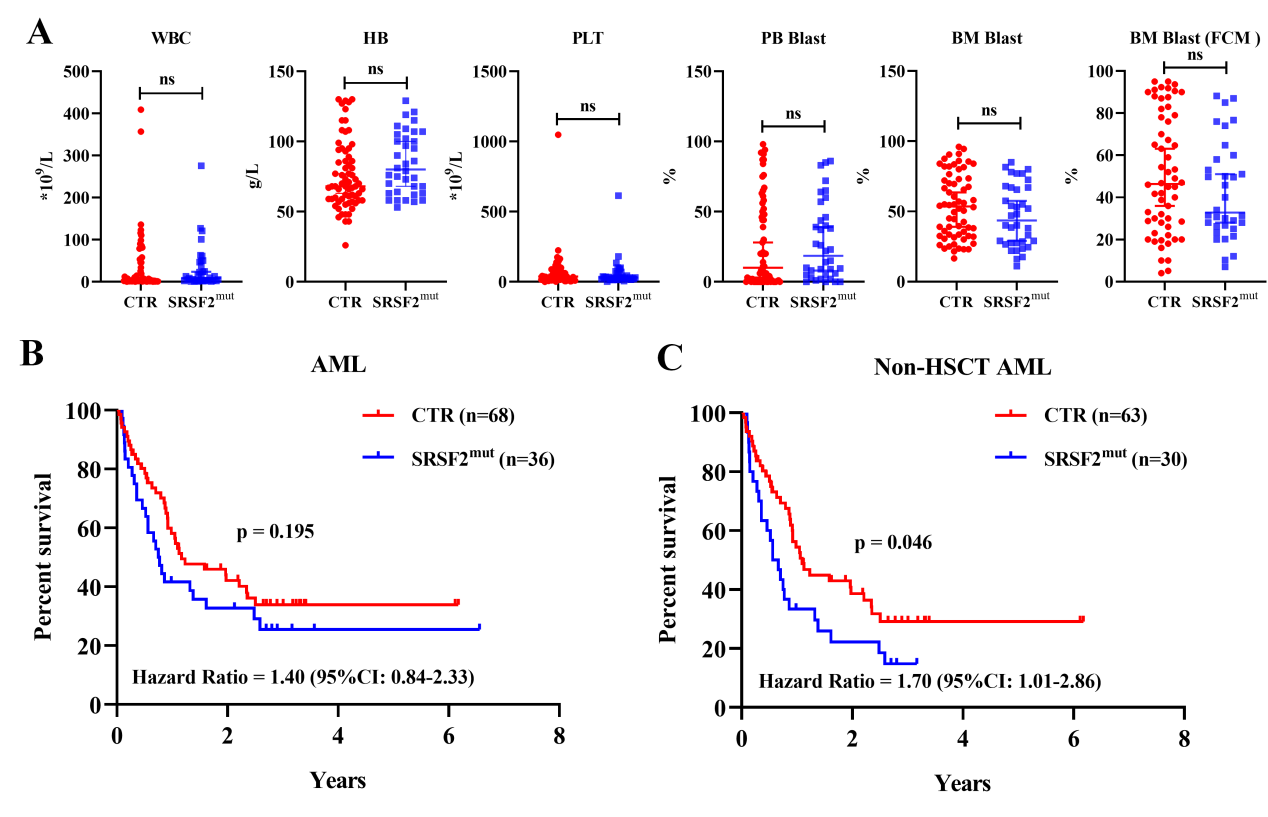


**Supplemental Figure 1** Comparative analysis of hematologic parameters, blast proportions, and survival outcomes between propensity score-matched cohorts. (A) Intergroup comparisons of WBC count, HB level, PLT count, PB blast percentage, BM blast proportion by morphological examination, and FCM-detected BM blast percentage. (B) Kaplan-Meier survival analysis of matched cohorts. (C) Survival analysis after censoring HSCT recipients

Abbreviations: WBC, white blood cell; HB, hemoglobin, PLT, platelet; PB, peripheral blood, BM, bone marrow; FCM, flow cytometry; HSCT, hematopoietic stem cell transplantation

**Supplemental Figure 2** Validation of *SRSF2* expression in HEL cells. (A) WB detection of SRSF2 and Flag-tagged protein expression. (B) qPCR analysis of SRSF2^mut^ transcript levels

Abbreviations: WB, Western blotting; qPCR, real-time quantitative polymerase chain reaction


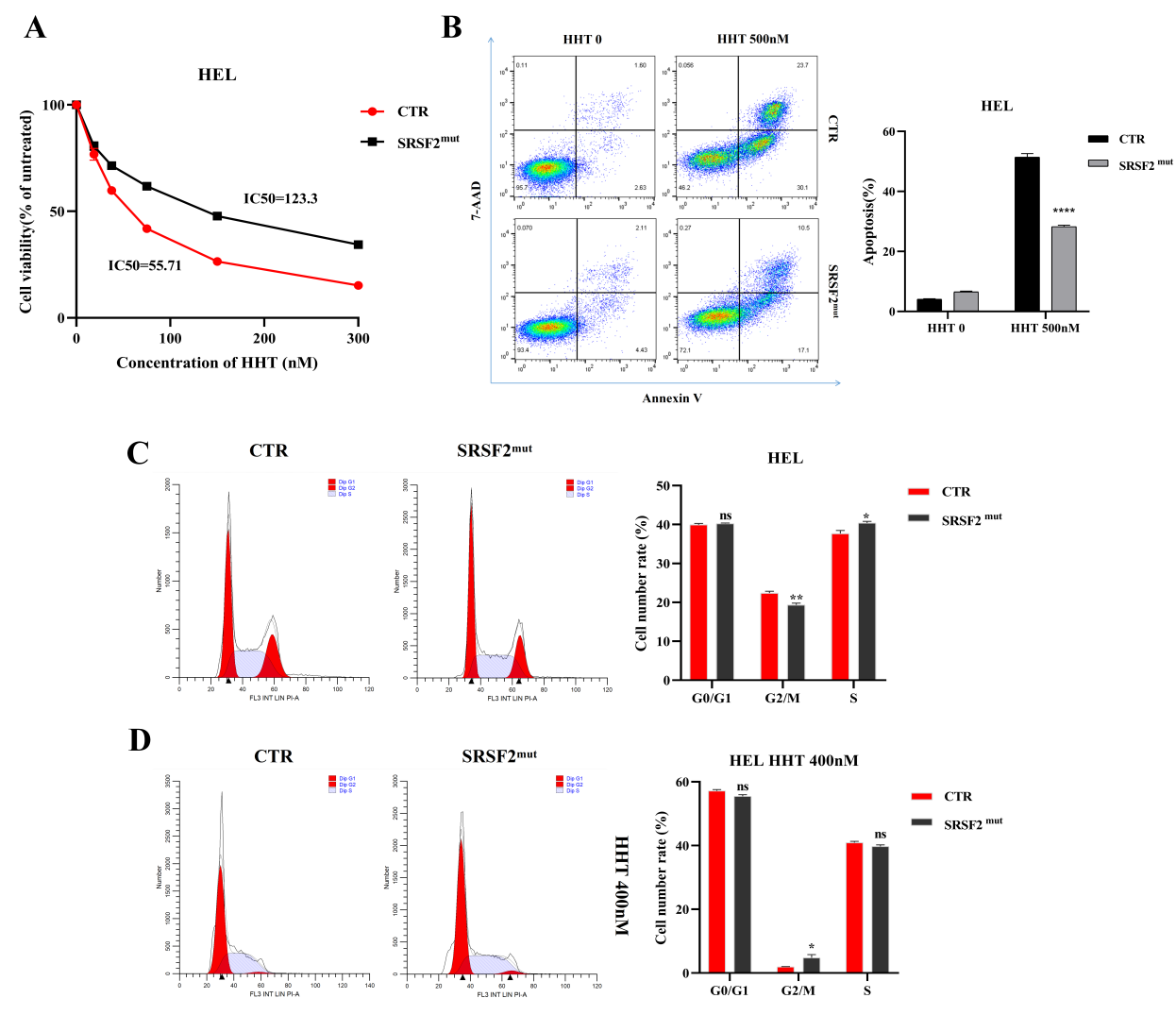


**Supplemental Figure 3** Effects of *SRSF2* mutation on cell cycle progression and HHT sensitivity in HEL cells. (A) Viability assessment of HHT-treated HEL cells. (B) Quantification of apoptosis in HHT-exposed HEL cells. (C) Impact of *SRSF2* mutation on the cell cycle distribution of HEL cells. (D) Cell cycle profiling of HEL cells after HHT exposure

Abbreviations: HHT, homoharringtonine


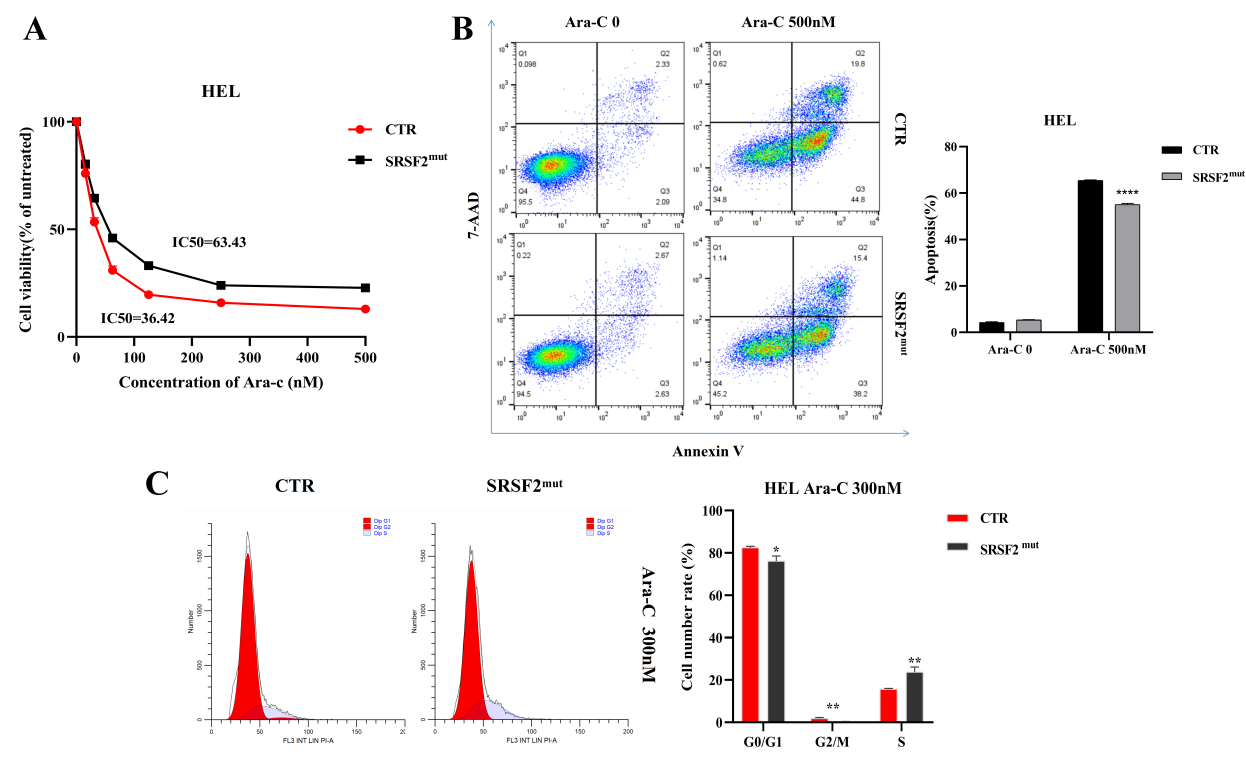


**Supplemental Figure 4** *SRSF2* mutation altered Ara-C sensitivity in HEL cells. (A) HEL cell viability upon Ara-C treatment. (B) Ara-C-induced apoptosis in HEL cells. (C) Cell cycle profiling of Ara-C-treated HEL cells

Abbreviations: Ara-C, cytarabine


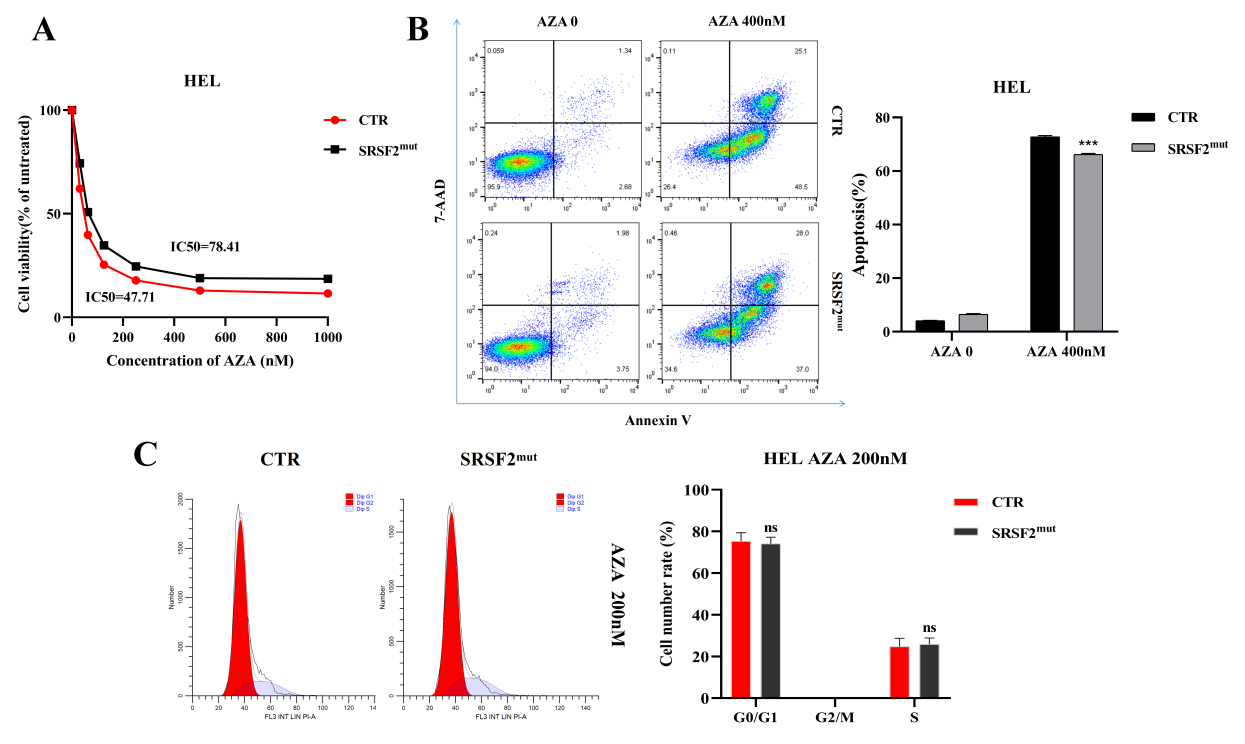


**Supplemental Figure 5** *SRSF2* mutation altered AZA sensitivity in HEL cells. (A) HEL cell viability upon AZA treatment. (B) AZA-induced apoptosis in HEL cells. (C) Cell cycle profiling of AZA-treated HEL cells

Abbreviations: AZA, azacitidine


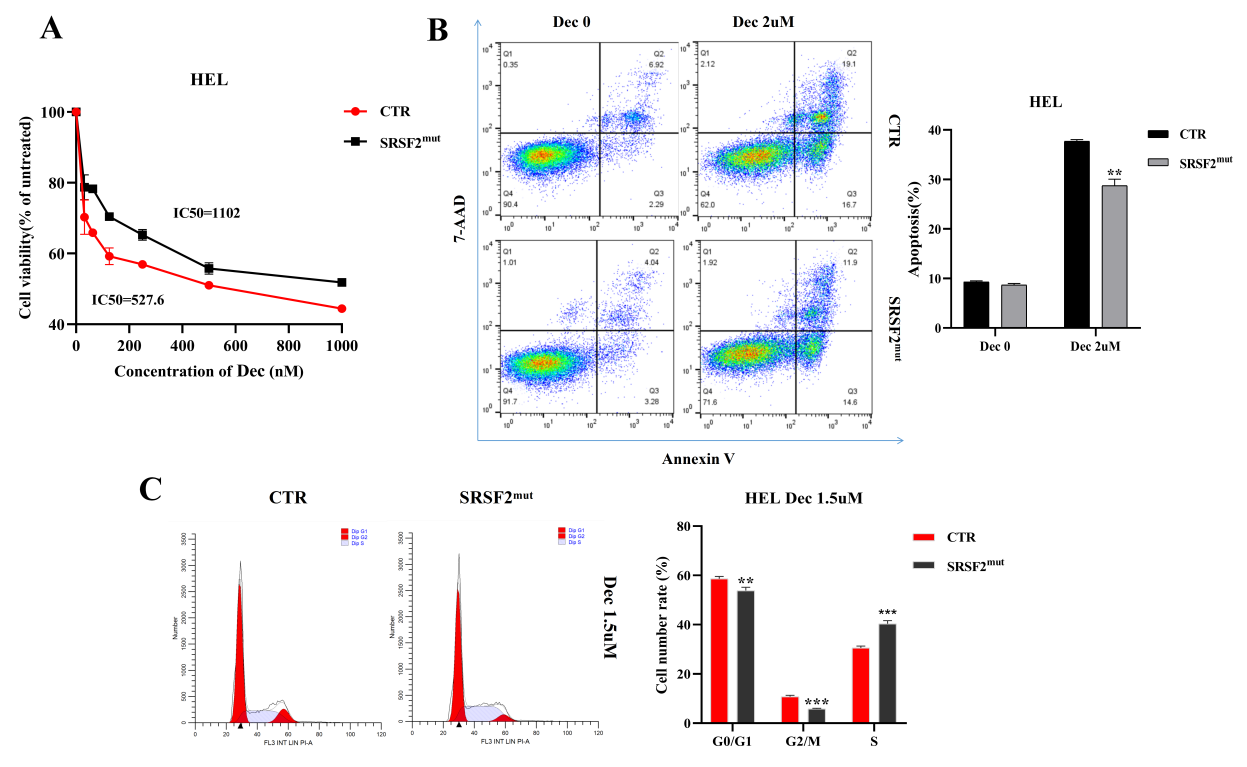


**Supplemental Figure 6** *SRSF2* mutation altered Dec sensitivity in HEL cells. (A) HEL cell viability upon Dec treatment. (B) Dec-induced apoptosis in HEL cells. (C) Cell cycle profiling of Dec-treated HEL cells

Abbreviations: Dec, decitabine


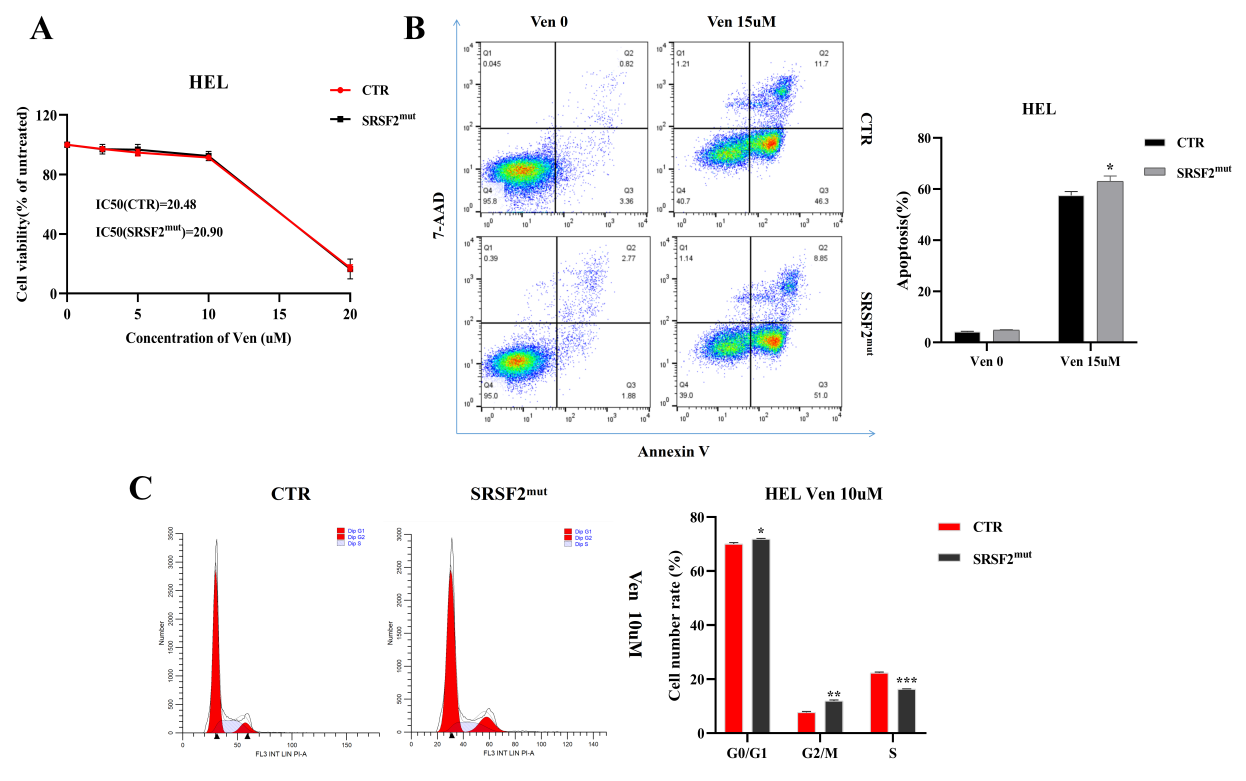


**Supplemental Figure 7** *SRSF2* mutation did not attenuate Ven sensitivity in HEL cells. (A) HEL cell viability upon Ven treatment. (B) Ven-induced apoptosis in HEL cells. (C) Cell cycle profiling of Ven-treated HEL cells

Abbreviations:Ven, venetoclax


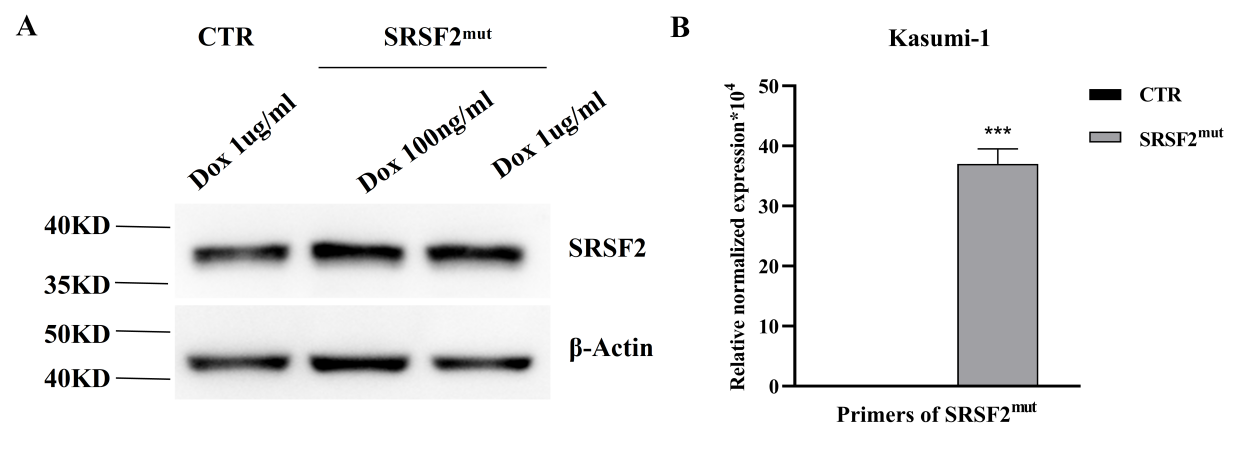


**Supplemental Figure 8** Validation of *SRSF2* expression in Kasumi-1 cells. (A) WB detection of the SRSF2 protein in CTR cells (1 μg/mL Dox) and SRSF2^mut^ cells (100 ng/mL or 1 μg/mL Dox) after 72 hours of induction. (B) qPCR analysis of SRSF2^mut^ transcript levels in Kasumi-1 cells after 72 hours of Dox induction (1 μg/mL)

Abbreviations: WB, Western blotting; CTR, control; Dox, doxycycline hyclate; qPCR, real-time quantitative polymerase chain reaction


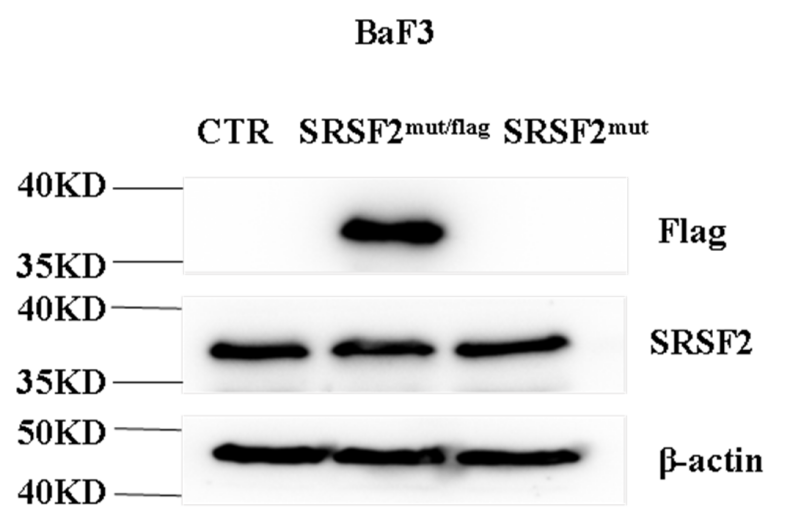


**Supplemental Figure 9** Western blotting detection of SRSF2 and Flag-tagged protein expression


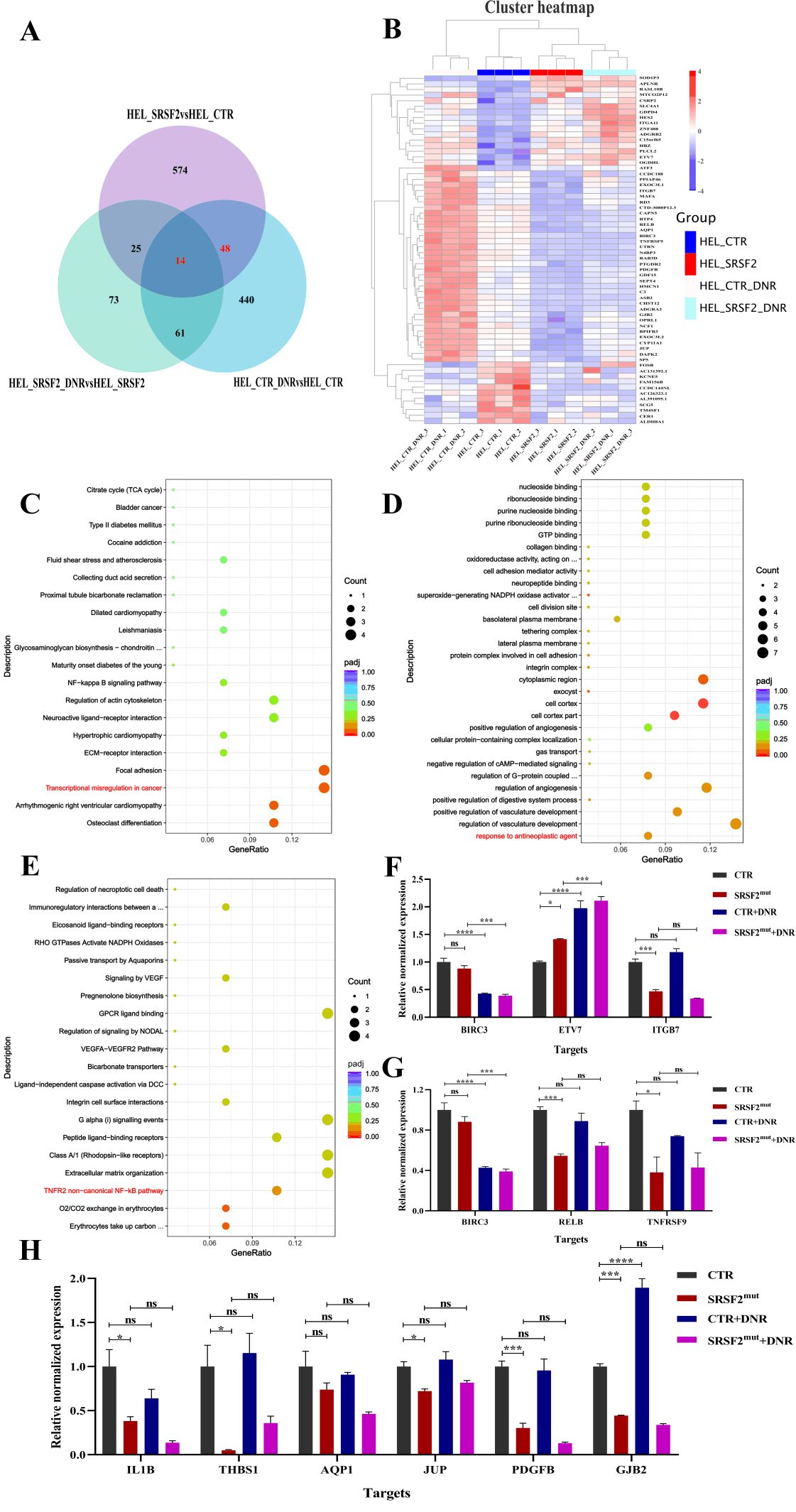


**Supplemental Figure 10** Integrated enrichment analysis and qPCR validation of DEGs in HEL cells. (A) Intersection analysis of DEGs via a Venn diagram. (B) Hierarchical clustering of highlighted genes from Venn analysis. (C) KEGG pathway enrichment analysis. (D) Gene Ontology enrichment analysis. (E) Reactome pathway analysis. (F-H) qPCR validation of DEGs in HEL cells

Abbreviations: qPCR, real-time quantitative polymerase chain reaction; DEGs, differentially expressed genes; KEGG, Kyoto Encyclopedia of Genes and Genomes


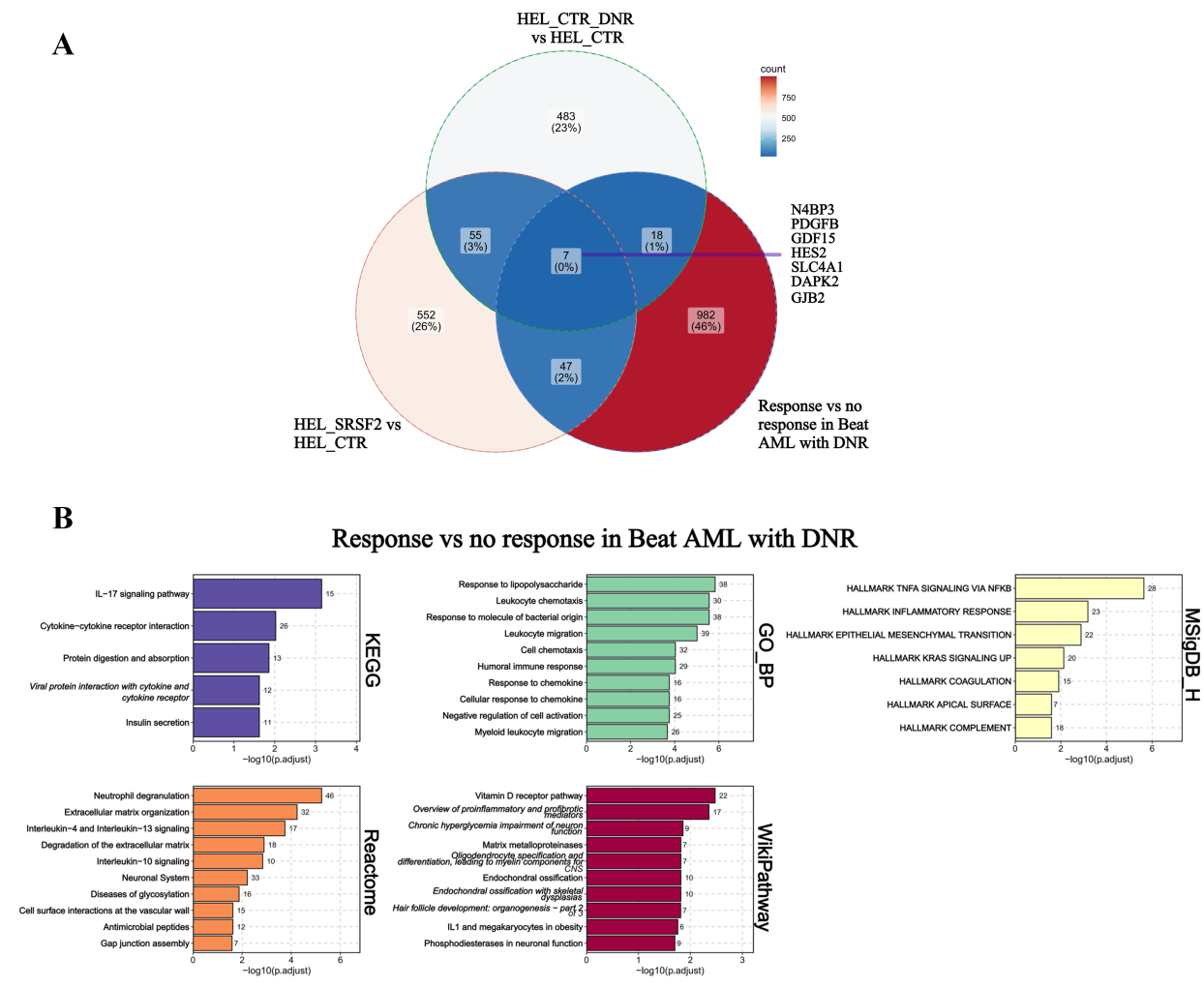


**Supplemental Figure 11** Comparative genomic analysis of the Beat AML dataset. (A) Venn diagram comparing differentially expressed genes between HEL cell groups and DNR-treated responsive/nonresponsive cases in the Beat AML dataset. (B) Enrichment analysis of the Beat AML dataset

Abbreviations: AML, acute myeloid leukemia; DNR, daunorubicin hydrochloride


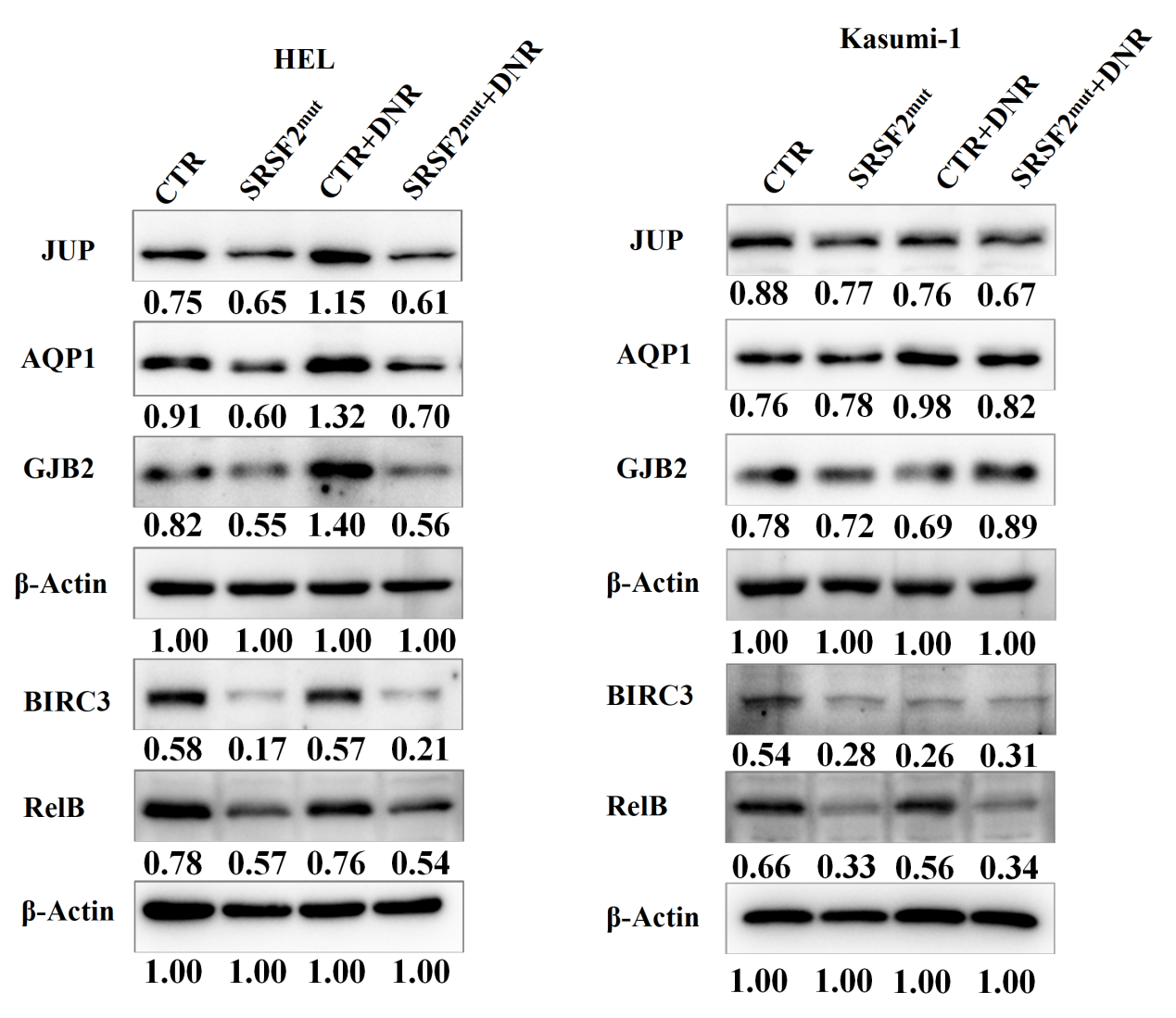


**Supplemental Figure 12** The protein expression levels of the genes were detected by WB. (A) WB detection of protein expression in HEL cells after 16 hours of treatment with 1x PBS or DNR. (B) WB analysis of protein levels in Kasumi-1 cells following 16 hours of exposure to 1x PBS or DNR

Abbreviations: WB, western blotting; PBS, phosphate-buffered saline; DNR, daunorubicin hydrochloride


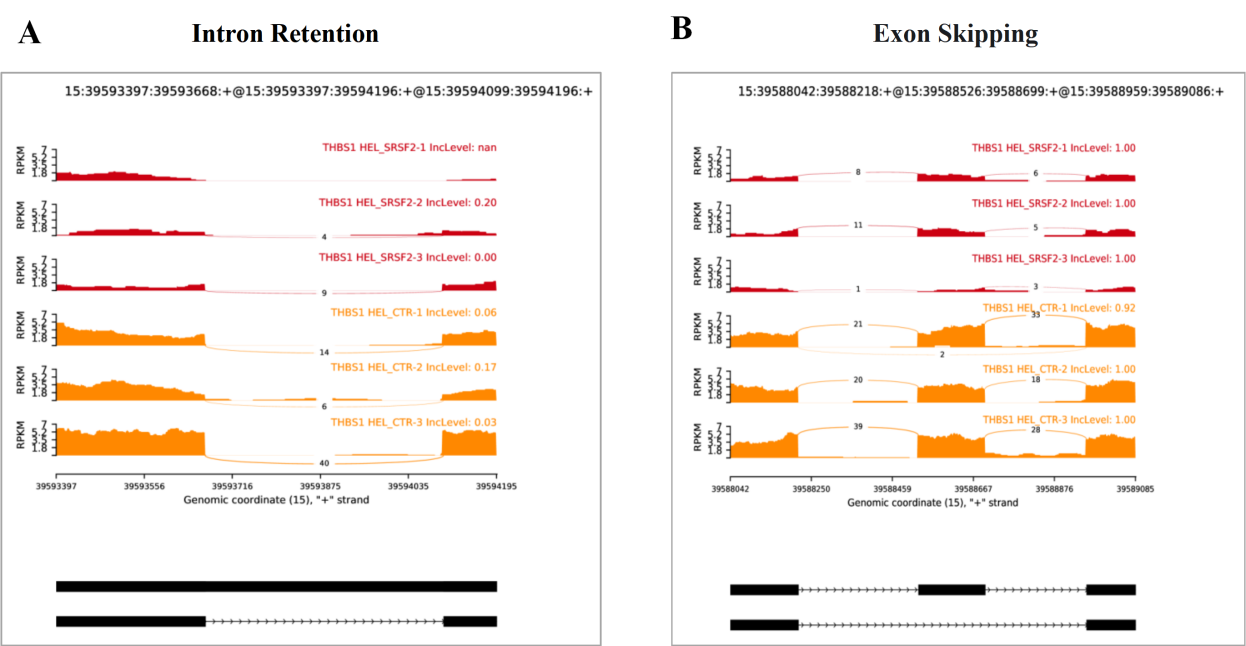


**Supplemental Figure 13** *SRSF2* mutation did not alter *THBS1* alternative splicing in HEL cells. (A) Intron retention. (B) Exon skipping


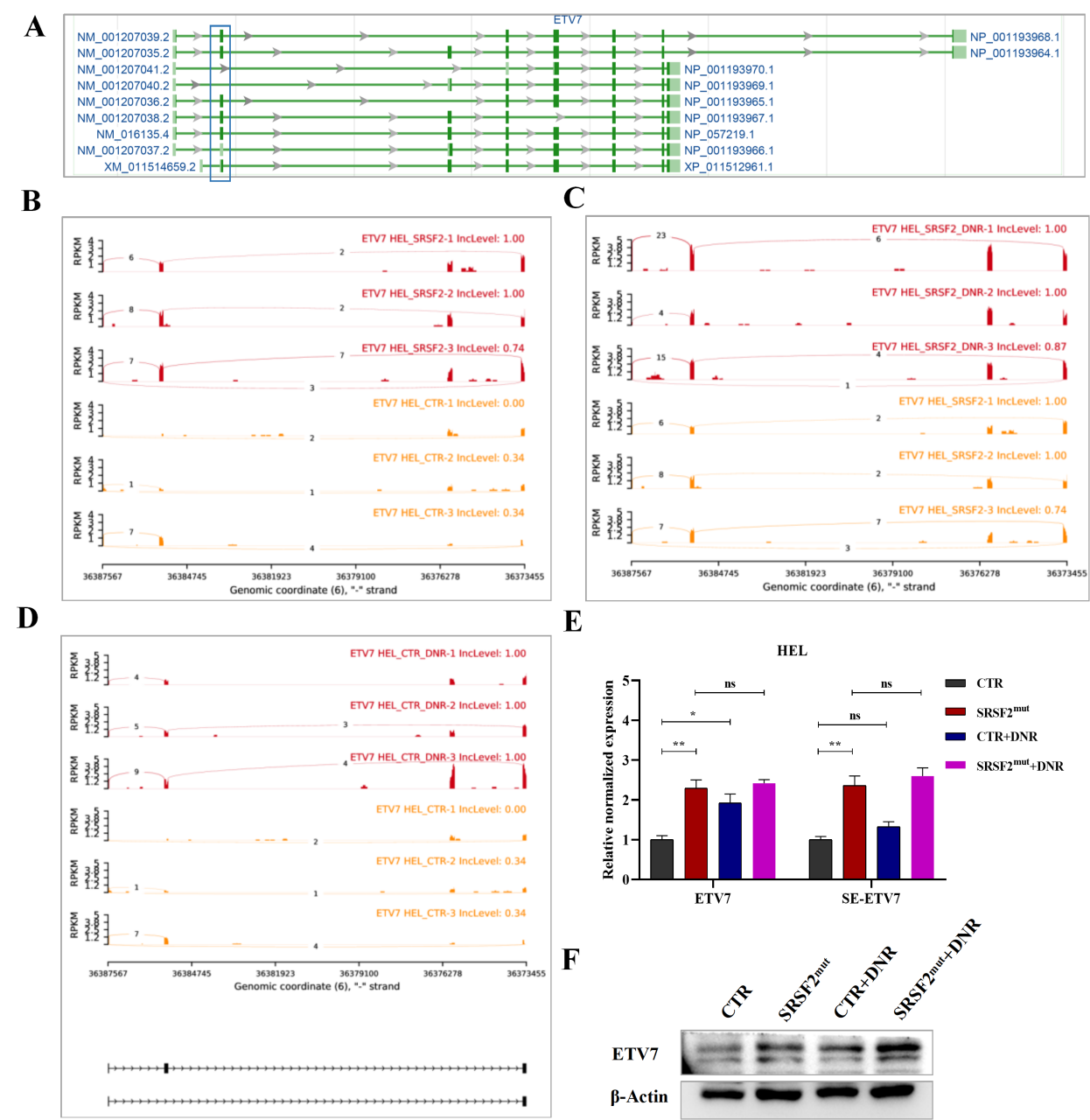


**Supplemental Figure 14** *SRSF2* mutation modulated *ETV7* splicing and expression. (A) Genomic annotation of *ETV7* variants (NCBI) highlighting exon 2. (B) *SRSF2* mutation-mediated alterations in *ETV7* exon skipping. (C) DNR-induced exon skipping dynamics in SRSF2^mut^ cells. (D) DNR-dependent splicing modulation in CTR cells. (E) Quantitative profiling of *ETV7* transcripts (total and exon 2-retained). (F) ETV7 proteomic validation by immunoblotting

Abbreviations: DNR, daunorubicin hydrochloride; CTR, control


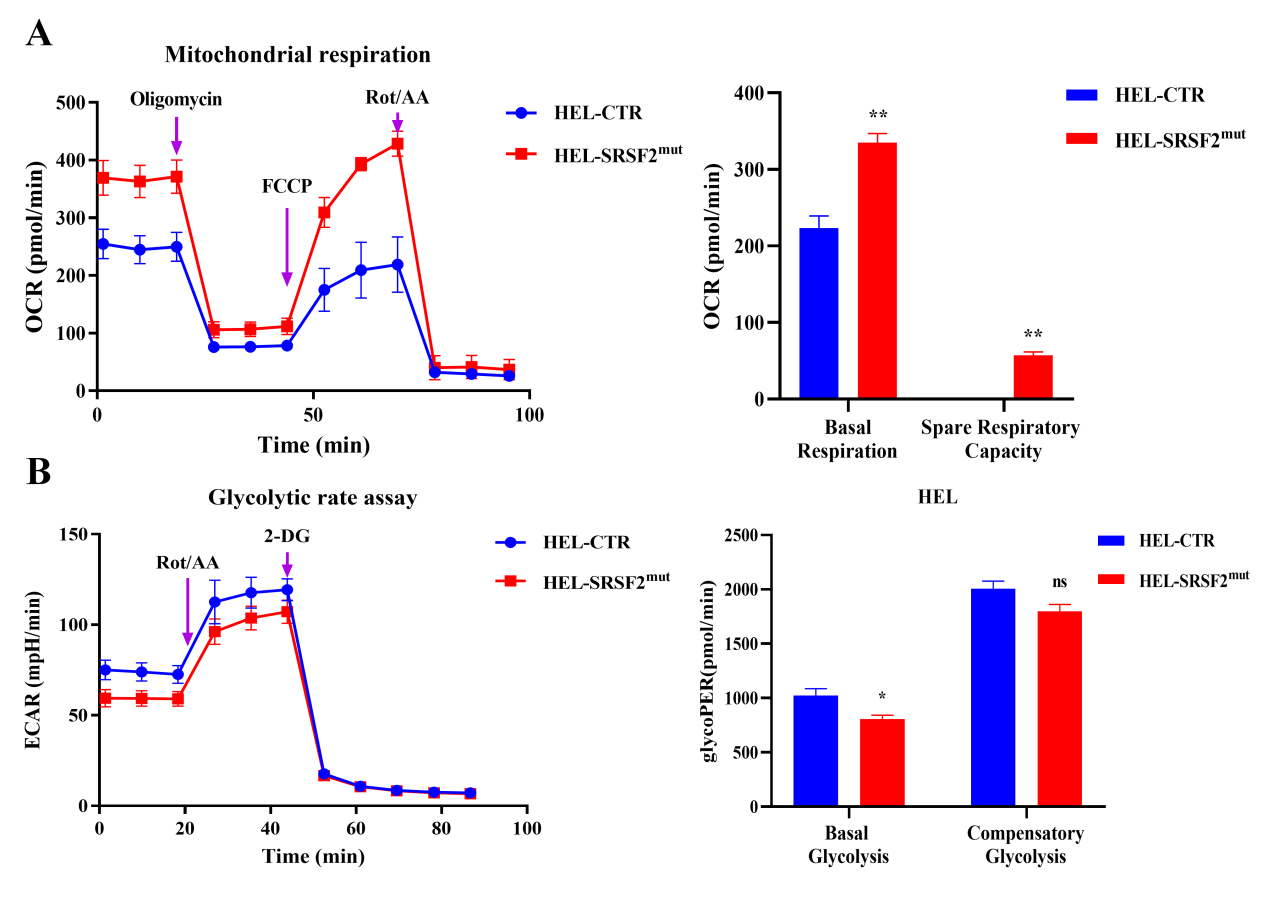


**Supplemental Figure 15** *SRSF2* mutation altered cellular metabolism. (A) Real-time OCR measurements during the mitochondrial stress assay in HEL cells. (B) Real-time ECAR measurements during the glycolytic rate assay

Abbreviations: OCR, oxygen consumption rate; ECAR, extracellular acidification rate


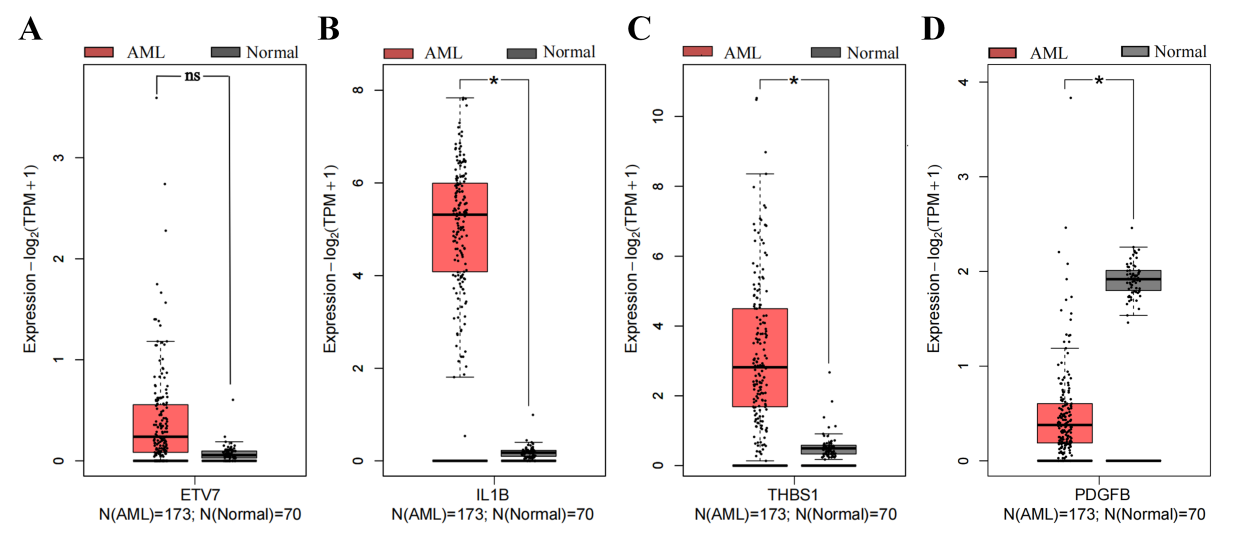


**Supplemental Figure 16** Differential expression analysis of *ETV7*, *IL1B*, *THBS1*, and *PDGFB* in normal tissues versus AML samples via GEPIA. (A) *ETV7*. (B) *IL1B*. (C) *THBS1*. (D) *PDGFB*

Abbreviations: AML, acute myeloid leukemia


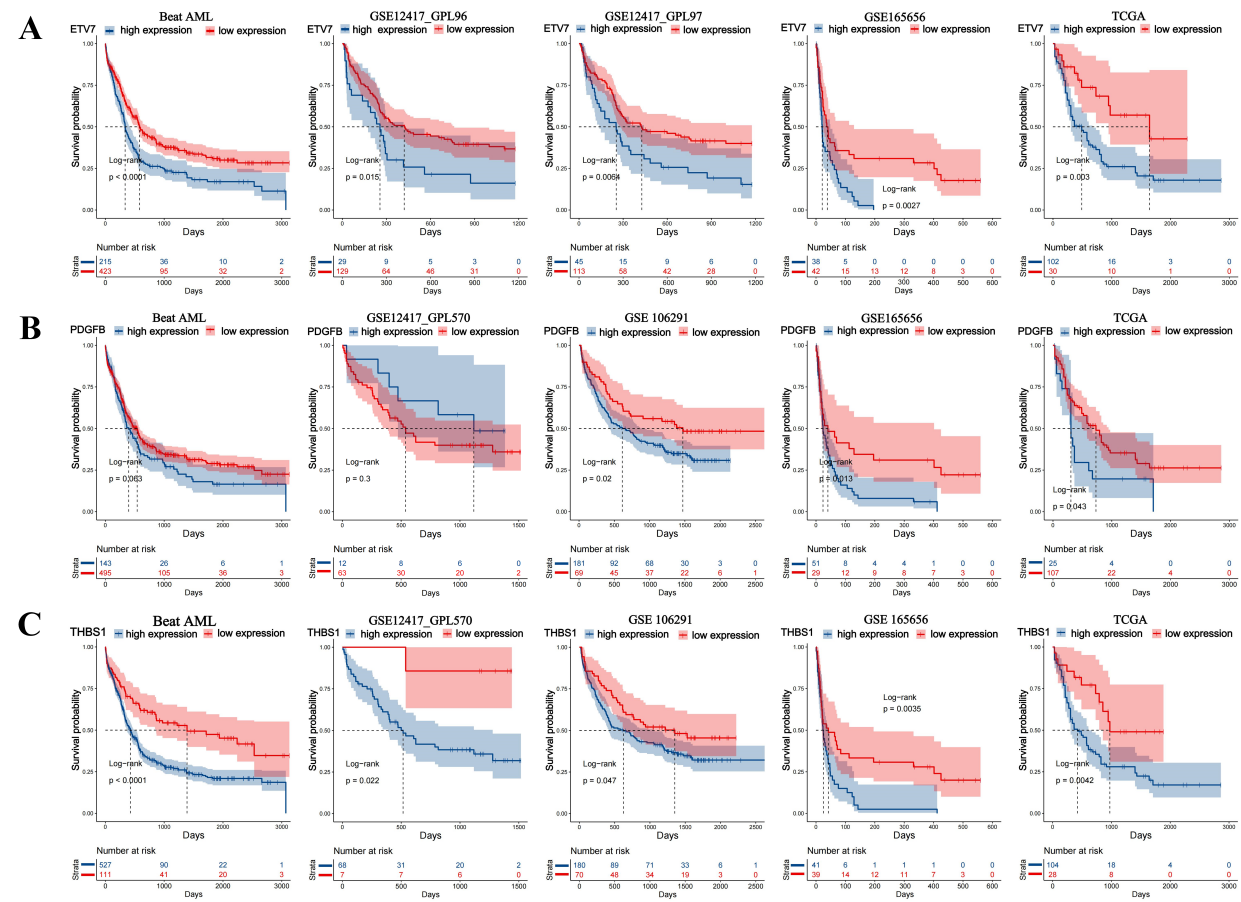


**Supplemental Figure 17** Prognostic value of altered *ETV7*, *PDGFB*, and *THBS1* expression patterns in AML patients through public database analysis. (A) *ETV7*. (B) *PDGFB*. (C) *THBS1*

Abbreviations: AML, acute myeloid leukemia
