# Supplementary material for: SRSF2 mutations drive daunorubicin resistance in acute myeloid leukemia via THBS1 stabilization
Source: J Exp Clin Cancer Res. 2026 Jan 30;45:64. doi: 10.1186/s13046-026-03649-y (PMC12973616; doi:10.1186/s13046-026-03649-y)
Supplement: Supplementary file 2 — Supplementary Material 2. [file 13046_2026_3649_MOESM2_ESM.docx]

**Supplemental Table 1** Sequences of primers used in this study

| Genes | Forward primers (5'→3') | Reverse primers (5'→3') |
| --- | --- | --- |
| *BIRC3* | GACTGGGCTTGTCCTTGCT | AGAAGTCGTTTTCCTCCTTTGT |
| *ETV7* | CGTGCAAGCCAGATGTGAAG | GTTCATCTCGAACCCGTGCT |
| *ITGB7* | GCCAGAGGAAGGACTGCTC | TCCCAAGCCGTAGTGGTAGA |
| *AQP1* | GCCTCTCTGTAGCCCTTGGA | TATTTGGGCTTCATCTCCACCC |
| *JUP* | CTCAGTTCGCTGTCCGC | TCAGTCACCTTGATAGGCTGC |
| *PDGFB* | CCGGAGTCGGCATGAATCG | CGGGTCATGTTCAGGTCCAA |
| *GJB2* | GCAGAGCAAACCGCCCA | AAAGTCGGCCTGCTCATCTC |
| *RELB* | CGTGCATGCTTCGGTCTG | TGATCTCCAATTCATCTGTGCT |
| *TNFRSF9* | TCTTCCTCACGCTCCGTTTC | AAGAAAGTCCCAACAGCCCT |
| *IL1B* | TTCGAGGCACAAGGCACAA | CCATCATTTCACTGGCGAGC |
| *THBS1* | AACCTCTACTCCGGACGCAC | CAGCAGGGATCCTGTGTGTA |

**Supplemental Table 2** Primers used for alternative splicing analysis

| Name | Forward primers (5'→3') | Reverse primers (5'→3') |
| --- | --- | --- |
| ETV7 | CGTGCAAGCCAGATGTGAAG | GTTCATCTCGAACCCGTGCT |
| S-ETV7 | GTTCTCGGAGCCAGGTTAGG | GCTTCACATCTGGCTTGCAC |

**Supplemental Table 3** Drug working solutions for the mitochondrial stress test

| Injection port | Compound | Final concentration (µM) | Stock volume (μL) | Assay medium volume (μL) | 10× Concentration (μM) | Loading volume (μL) |
| --- | --- | --- | --- | --- | --- | --- |
| A | Oligomycin | 1.5 | 1 | 999 | 15 | 56 |
| B | FCCP | 2.0 | 20 | 980 | 20 | 62 |
| C | Rot/AA | 0.5 | 1 | 999 | 5 | 69 |

**Supplemental Table 4** Drug working solutions for the glycolytic rate assay

| Injection port | Compound | Final concentration (µM) | Stock volume (μL) | Assay medium volume (μL) | 10× Concentration (μM) | Loading volume (μL) |
| --- | --- | --- | --- | --- | --- | --- |
| A | Rot/AA | 0.5 | 1 | 999 | 5 | 56 |
| B | 2-DG | 50 | 1000 | 0 | 500 | 62 |

**Supplemental Table 5** Baseline characteristics of the propensity score-matched cohort

|  | level | Control | SRSF2^mut^ | *P* value | SMD |
| --- | --- | --- | --- | --- | --- |
| n |  | 68 | 36 |  |  |
| sex (%) | male | 46 (67.6) | 25 (69.4) | 1 | 0.039 |
|  | female | 22 (32.4) | 11 (30.6) |  |  |
| age (%) | ~17 | 0 (0.0) | 0 (0.0) | NA | 0.013 |
|  | 18~44 | 2 (2.9) | 1 (2.8) |  |  |
|  | 45~59 | 21 (30.9) | 11 (30.6) |  |  |
|  | 60 ~ | 45 (66.2) | 24 (66.7) |  |  |
| *NPM1* (%) | wild type | 63 (92.6) | 33 (91.7) | 1 | 0.036 |
|  | mutant | 5 (7.4) | 3 (8.3) |  |  |
| *CEBPA* biallelic (%) | wild type | 66 (97.1) | 35 (97.2) | 1 | 0.01 |
|  | mutant | 2 (2.9) | 1 (2.8) |  |  |
| *FLT3*-ITD (%) | wild type | 67 (98.5) | 35 (97.2) | 1 | 0.091 |
|  | mutant | 1 (1.5) | 1 (2.8) |  |  |
| *TP53* (%) | wild type | 64 (94.1) | 34 (94.4) | 1 | 0.014 |
|  | mutant | 4 (5.9) | 2 (5.6) |  |  |
| *GATA2* (%) | wild type | 66 (97.1) | 35 (97.2) | 1 | 0.01 |
|  | mutant | 2 (2.9) | 1 (2.8) |  |  |
| *BCOR* (%) | wild type | 62 (91.2) | 33 (91.7) | 1 | 0.018 |
|  | mutant | 6 (8.8) | 3 (8.3) |  |  |
| *SF3B1* (%) | wild type | 64 (94.1) | 34 (94.4) | 1 | 0.014 |
|  | mutant | 4 (5.9) | 2 (5.6) |  |  |
| *EZH2* (%) | wild type | 66 (97.1) | 36 (100.0) | 0.773 | 0.246 |
|  | mutant | 2 (2.9) | 0 (0.0) |  |  |
| *ZRSR2* (%) | wild type | 66 (97.1) | 36 (100.0) | 0.773 | 0.246 |
|  | mutant | 2 (2.9) | 0 (0.0) |  |  |
| *U2AF1* (%) | wild type | 62 (91.2) | 36 (100.0) | 0.163 | 0.44 |
|  | mutant | 6 (8.8) | 0 (0.0) |  |  |
| *STAG2* (%) | wild type | 61 (89.7) | 27 (75.0) | 0.091 | 0.393 |
|  | mutant | 7 (10.3) | 9 (25.0) |  |  |
| *RUNX1* (%) | wild type | 60 (88.2) | 24 (66.7) | 0.017 | 0.534 |
|  | mutant | 8 (11.8) | 12 (33.3) |  |  |
| *ASXL1* (%) | wild type | 59 (86.8) | 16 (44.4) | <0.001 | 0.995 |
|  | mutant | 9 (13.2) | 20 (55.6) |  |  |

Statistical note: A *P* value threshold of ≥0.05 was used to indicate no statistically significant differences between propensity score-matched groups for any given variable. Between-group balance was quantitatively assessed via standardized mean differences (SMDs), where smaller absolute values denote better covariate balance
